# Comparing nitisinone 2 mg and 10 mg in the treatment of alkaptonuria—An approach using statistical modelling

**DOI:** 10.1002/jmd2.12261

**Published:** 2021-11-11

**Authors:** Lakshminarayan R. Ranganath, Anna M. Milan, Andrew T. Hughes, Milad Khedr, Brendan P. Norman, Mohammed Alsbou, Richard Imrich, Matthew Gornall, Nicolas Sireau, James A. Gallagher, Richard Jackson

**Affiliations:** ^1^ Departments of Clinical Biochemistry and Metabolic Medicine Liverpool University Hospitals NHS Foundation Trusts Liverpool UK; ^2^ Musculoskeletal Biology and Ageing University of Liverpool, William Henry Duncan Building Liverpool UK; ^3^ Faculty of Medicine Mutah University Karak Jordan; ^4^ National Institute of Rheumatic Diseases Piešťany Slovakia; ^5^ Biomedical Research Center, Slovak Academy of Sciences Bratislava Slovakia; ^6^ Liverpool Cancer Trials Unit University of Liverpool Liverpool UK; ^7^ AKU Society Cambridge UK

**Keywords:** AKUSSI, alkaptonuria, disease progression, homogentisic acid, nitisinone, safety, slope analysis

## Abstract

**Background:**

Outcomes from studies employing nitisinone 10 mg and 2 mg in alkaptonuria were compared.

**Patients and methods:**

Sixty‐nine patients in each of the nitisinone (10 mg daily) and controls of suitability of nitisinone in alkaptonuria 2 (SONIA 2), as well as 37 and 23 in nitisinone (2 mg daily) and control cohorts at the National Alkaptonuria Centre (NAC), respectively, were followed up for 4 years. Severity of alkaptonuria (AKU) was assessed by the AKU Severity Score Index (AKUSSI). 24‐h urine homogentisic acid (uHGA_24_), serum HGA (sHGA), serum tyrosine (sTYR) and serum nitisinone (sNIT) were also analysed at each time point. Dietetic support was used in the NAC, but not in SONIA 2. Safety outcomes were also compared. All statistical analyses were post hoc.

**Results:**

The slope of the AKUSSI was 0.55, 0.19, 0.30, and 0.06 per month in the control NAC, nitisinone NAC, control SONIA 2, and nitisinone SONIA 2 cohorts, respectively. The intersection of the slopes on the *x*‐axis was −132, −411, −295, and − 1460 months, respectively. The control and nitisinone slope comparisons were statistically significant both in the NAC (*p* < 0.001) and the SONIA 2 (*p* < 0.001). Corneal keratopathy occurred in 3 and 10 patients in the NAC and SONIA 2, respectively.

**Discussion:**

The nitisinone 10 mg dose decreased disease progression more than the 2 mg dose although the incidence of corneal keratopathy was 14.5% and 4.9%, respectively.

**Conclusion:**

Nitisinone 10 mg decreased urine and serum HGA, increased serum tyrosine, and decreased disease progression more than 2 mg. Low‐protein dietetic support may be needed to mitigate tyrosinaemia following nitisinone.

**Highlights:**

1. Nitisinone 10 mg apparently slows alkaptonuria disease progression more than 2 mg in adults.
2. Corneal keratopathy during nitisinone therapy was more common in men.
3. Serum nitisinone concentrations increased significantly over time.
4. Nitisinone may inhibit cytochrome P450 self catabolism.

## 1 INTRODUCTION

Alkaptonuria (AKU; OMIM#203500)^1^, ^2^ is an autosomal recessive genetic disorder characterised by deficiency of homogentisate dioxygenase activity (EC:1.13.11.5), with a prevalence of around 1 in 250 000 in most non‐consanguineous societies. The inability to fully metabolise ingested dietary phenylalanine and tyrosine results in accumulation of homogentisic acid (HGA).^3^ The increase in circulating HGA is directly causal in the disease process, known as ochronosis, in which a melanin‐like HGA pigment is deposited in connective tissues including cartilage, causing these tissues to break down.^4^ Manifestations of AKU include premature arthritis, cardiac valve disease, fractures, and ruptures of muscle and tendon. Significant amounts of accumulating HGA is excreted in the urine and can lead not only to dark urine but also to renal stones.^5^

Until recently, there was a lack of HGA‐lowering disease‐modifying therapy.^6^ In 1998, it was suggested that nitisinone, already in use as treatment of hereditary tyrosinaemia type 1 (HT‐1; OMIM 276700),^7^ could also decrease HGA.^8^ Nitisinone inhibits the enzyme p‐hydroxyphenylpyruvate dioxygenase (HPPD) (EC:1.13.11.27), thereby decreasing the accumulation of HGA.^2^, ^9^, ^10^ Ochronotic pigment develops from HGA; therefore, a decrease in HGA could decrease pigment, and consequently the morbidity of AKU. Initial studies while not carrying out a full dose–response study of nitisinone in AKU arrived at an oral dose of 2 mg daily, adopted for the first nitisinone interventional study^10^; 20 nitisinone‐treated patients were compared with 20 controls, employing lateral rotation of the hip as the primary outcome, with the study reporting inconclusive. Subsequently, the United Kingdom National Health Service England Highly Specialised Services (HSS) commissioned a service, the National Alkaptonuria Centre (NAC) in the Royal Liverpool University Hospital (RLUH), where off‐label use of oral nitisinone 2 mg daily has been in use since 2012. The NAC, unlike the National Institutes of Health (NIH) trial, employs a composite disease severity score called the AKU Severity Score Index (AKUSSI) to determine outcomes.^11^ The NAC reported that nitisinone decreased HGA, reduced visible ochronosis in eyes and ears, and arrested progression of this complex multisystem, slowly progressive disorder. More recently, a 4‐year randomised multicentre international clinical study, called suitability of nitisinone in alkaptonuria 2 (SONIA 2), replicated the results of the off‐label use of nitisinone in the NAC, using a daily dose of 10 mg, which has since been approved as the first disease‐modifying treatment of adult AKU by the European Medicines Agency and the European Commission.^12^

There is a cost to administration of lifelong nitisinone therapy, which is dependent on the dose of nitisinone used.^13^ Therefore, the analysis of the efficacy and safety of nitisinone 2 versus 10 mg daily dose needs to be carried out. A direct comparison of nitisinone 2 and 10 mg in a clinical study is not realistic, although there is published evaluation of these doses over the short term.^14^

As in SONIA 2, protocolised data were collected in the off‐label use of nitisinone in the NAC^15^; high‐quality data were therefore collected in the NAC similar to what was done in SONIA 2. This allows comparison of efficacy and safety data from these two data sets. The objective of this manuscript is to compare the efficacy and specific aspects of safety of nitisinone 2 mg daily with 10 mg daily, in order to guide nitisinone dosing in AKU, an issue also raised in a recently published communication.^16^

## 2 METHODS

### 2.1 Patients

#### 2.1.1 The NAC cohort

Patients with confirmed AKU, through documented increase in urine HGA (uHGA), attended the RLUH between 2007 and 2020. Attendance between 2007 and 2011, a single non‐interventional visit (VR), was part of a research study (Natural History study in alkaptonuria; UK Research Ethics Committee Number 07/Q1002/111).^11^, ^15^ Some of these patients constitute the control group. The NAC provides a comprehensive service including off‐label nitisinone. No patient received nitisinone prior to 2012. Patients who started attending the NAC after June 2012 are only included in the nitisinone group. The visits were designated as V0, V1, V2, V3, and V4, to denote baseline, 12, 24, 36, and 48 months in the study. The plan of the visits and numbers of patients are shown in Figure S1A, and this is, along with ethics and consent considerations, are described in more detail in the Supplementary Material. V0 was the baseline visit, and the duration between VR and V0 varied among control patients as the data were collected as part of providing a service. The 4‐year nitisinone data from the NAC were chosen to allow a direct comparison with SONIA 2 which had a duration of 4 years.

#### 2.1.2 The SONIA 2 study cohort

SONIA 2 was a 4‐year, open‐label, evaluator‐blinded, multicentre, randomised, no‐treatment controlled, parallel‐group study described further in the Supplementary Material (Figure S1B). The study sites were Liverpool (UK), Paris (France), and Piešťany (Slovakia). The aim was to recruit 140 patients, 70 in control and 70 randomised to nitisinone 10 mg, aged 25 years or older, with diagnosed AKU and any clinical manifestation in addition to increased HGA. The visits were designated as V0, V3, V12, V24, V36, and V48, to denote baseline, 3, 12, 24, 36, and 48 months in the study; this was named differently from NAC due to the extra 3‐month visit. Patients who developed keratopathy were put on 2 mg nitisinone after temporary withdrawal and recovery. Independent ethics committees at each centre approved the study. All patients provided written informed consent prior to inclusion in the NAC and SONIA 2 cohorts.

#### 2.1.3 Assessment at every visit

The AKUSSI is a semi‐quantitative composite score that collects data from a large number of assessments to calculate the severity of AKU, which, by not relying on a single disease feature, increases the likelihood of detecting a treatment effect.^17^ The AKUSSI items include the morbid features assessed by a variety of assessments including historical data and investigations. The NAC and SONIA 2 both employed the AKUSSI to determine the effect of nitisinone on disease progression.

#### 2.1.4 Management of tyrosinaemia following nitisinone therapy in the NAC and SONIA 2

At the NAC, a specialist dietician managed the increase in serum tyrosine, to defined serum tyrosine thresholds (Table S1), employing 7‐day food diaries and blood spot serum tyrosine remote monitoring. The aim was to keep serum tyrosine (sTYR) below 500 μmol/L. In SONIA 2, the diet of patients was not actively managed, apart from providing information sheets regarding eating a lower protein diet. Details of how corneal keratopathy was managed in terms of assessment and nitisinone treatment are described in the Supplementary Material.

#### 2.1.5 Chemical and data analyses

In both studies, measurements of 24‐huHGA_24_ and fasting serum for HGA (sHGA), tyrosine (sTYR), and nitisinone (sNIT) were carried out. Other assessments included a range of clinical outcome measures (AKUSSI), safety assessment, and other procedures as described elsewhere.^11^, ^15^ There were minor differences in the AKUSSI scoring in the NAC and the SONIA 2 cohorts (Table S2A,B). However, the AKUSSI scoring of control and nitisinone comparisons within each of these two cohorts were identical.

#### 2.1.6 Statistical analysis

Continuous variables for metabolites uHGA_24_, sHGA, sTYR, and sNIT were compared using ANOVA with Tukey–Kramer test for multiple comparisons using Instat GraphPad 3. Two‐sided 95% confidence intervals corresponding to a two‐sided 5% level of significance were used throughout the analyses. SONIA 2 analyses described here were post hoc.

The progression of the AKU disease was calculated using linear regression of AKUSSI versus time to obtain the average slope of the change in AKUSSI.^18^, ^19^, ^20^ Models also included centred variables for age and gender to account for any effect of these demographics. The analyses were conducted for each of the four data sets separately. Backwards extrapolation based on the model slope parameters were performed to estimate the point in past time at which AKUSSI is zero, that is, a hypothetical point just before disease features could have started. In terms of the analysis of slopes and intersection, data are presented in terms of medians (interquartile range). All analyses were conducted using a *p* < 0.05 to determine statistical significance, using R (Version 4.0).

#### 2.1.7 Role of the funding source

The funding sources were not involved in the study design, collection, analysis and interpretation of data, the writing of the manuscript, or in the decision to submit the manuscript for publication.

## 3 RESULTS

### 3.1 Baseline demographic and other parameters in the NAC and SONIA 2

The comparison of changes in uHGA_24_, sHGA, sTYR, sNIT, and AKUSSI, within the controls and the nitisinone groups, as well as between the controls and the nitisinone groups, both in the NAC and the SONIA 2, can be found in previous publications.^11^, ^12^ However, for ease of access, relevant tables and figures containing these data are included in the Supplementary Material, but will not be discussed here, as the focus is on the direct comparison of the SONIA 2 and the NAC. In the NAC nitisinone group, there were 37 patients, 23 men and 14 women, with mean (±*SD*) age of 46·8 ± 14·5 years (Table 1). In the NAC control group, there were 23 patients, 15 men and 8 women, with a mean (±*SD*) age of 47·4 ± 14·8 years (Table 1). In the SONIA 2 nitisinone group, there were 69 patients, 45 men and 24 women; with mean (*SD*) age of 49·0 ± 11·3 years. The SONIA 2 control group consisted of 69 patients, 40 men and 29 women, with a mean age of 47·7 ± 10·2 years. uHGA_24_, sHGA, and sTYR tended to be higher in men than in women both in the NAC and SONIA 2.

**TABLE 1:** Baseline demographic and related variables in the NAC and SONIA 2 studies—Mean (*SD*)

	Control group			Nitisinone group		
	All	Male	Female	All	Male	Female
National Alkaptonuria Centre (NAC)						
Numbers of patients	23	15	8	37	23	14
Age (years)	47.4 (14.8)	44.3 (14.8)	53.4 (13.7)	46.8 (14.5)	42.8 (12.9)	53.4 (15.1)
Weight (kg)	75.1 (18)	83.1 (16.2)	60.1 (10)	74.4 (14)	80.4 (11.2)	64.6 (12.8)
Body mass index (kg/M^2^)	27 (5.2)	28.4 (5.4)	24.6 (4.1)	26.9 (4.1)	27.2 (3.6)	26.4 (4.9)
uHGA_24_ (μmol/day)	25 230 (13 973)	30 013 (14 807)	17 259 (9610)	21 845 (9557)	24 502 (10 361)	17 669 (6464)
sHGA (μmol/L)	28.3 (15.2)	35.1 (13.9)	17.0 (10.8)	26.2 (13.1)	26.1 (10)	26.5 (17.6)
sTYR (μmol/L)	51.4 (15.4)	59.2 (14.4)	38.3 (2.3)	53.9 (43.7)	59.4 (53.3)	44.6 (16.9)
Number of patients with keratopathy	0	0	0	3	3	0
SONIA 2						
Numbers of patients	69	40	29	69	45	24
Age (years)	47.7 (10.2)	48.1 (9.9)	47 (10.7)	49 (11.3)	47.4 (11.9)	51.9 (9.6)
Weight (kg)	74.1 (15.6)	80.4 (13.3)	65.6 (14.6)	74.8 (14.8)	79.2 (12.6)	66.3 (15.1)
Body mass index (kg/M^2^)	26.4 (4.6)	27 (4.1)	25.5 (5.2)	26.9 (4.4)	27.3 (4.2)	26.2 (4.7)
uHGA_24_ (μmol/day)	35 394 (13 868)	38 740 (12 282)	30 778 (14 797)	35 019 (13 124)	37 149 (12 583)	31 024 (13 447)
sHGA (μmol/L)	28.3 (8.7)	29.1 (7.7)	27.1 (9.8)	30.3 (11)	31.7 (11.2)	27.9 (10.4)
sTYR (μmol/L)	64.5 (15.5)	69.4 (15.3)	57.8 (13.1)	65.3 (14.8)	67 (13.7)	62.2 (16.6)
Number of patients with keratopathy	0	0	0	10	8	2

Abbreviations: NAC, National Alkaptonuria Centre; sHGA, serum homogentisic acid; SONIA 2, suitability of nitisinone in alkaptonuria 2; sTYR, serum tyrosine; uHGA_24_, 24‐h urine homogentisic acid.

### 3.2 Changes in uHGA_24_ at follow‐up in the NAC and SONIA 2

Changes in uHGA_24_ in the nitisinone and control groups in the NAC and SONIA 2 are shown in Figure S2A–D (Tables S3 and S4). Direct comparisons of baseline, 1‐, 2‐, 3‐, and 4‐year visits between SONIA 2 and NAC in the nitisinone group are shown in Table 2. uHGA_24_ was significantly higher at baseline but lower at the 1 year during nitisinone therapy in SONIA 2 compared with NAC.

**TABLE 2:** Direct comparison following nitisinone of uHGA24, sHGA, and sTYR concentrations at 12‐, 24‐, 36‐, and 48‐month visits in SONIA 2 and NAC—Mean (*SD*)

		Visits				
Measurements	Cohorts	Baseline	12 months	24 months	36 months	48 months
uHGA_24_ (μmol/day)	SONIA 2	35 019 (13 124)***	181 (401)***	990 (3870)	1640 (6365)	1542 (6166)
	NAC	21 845 (9557)	1249 (946)	1328 (1187)	1153 (847)	1218 (978)
sHGA (μmol/L)	SONIA 2	30.3 (11.0)	0.74 (1.67)***	2.07 (6.55)*	2.49 (6.36)**	2.23 (5.99)**
	NAC	26.2 (13.1)	4.33 (1.65)	4.42 (2.93)	3.47 (0.97)	3.86 (2.2)
sTYR (μmol/L)	SONIA 2	65.3 (14.8)	918 (205)***	884 (230)***	894 (285)	875 (305)
	NAC	53.9 (43.7)	707 (153)	701 (211)	799 (156)	782 (181)
sNIT (μmol/L)	SONIA 2		4.34 (1.86)***	5.04 (2.67)***	5.28 (2.74)***	5.91 (3.92)***
	NAC		1.26 (0.64)	0.92 (0.41)	1.02 (0.34)	1.03 (0.67)

*Note*: *p < 0.05; **p < 0.01; ***p < 0.001 for SONIA 2 versus NAC comparisons.

Abbreviations: NAC, National Alkaptonuria Centre; sHGA, serum homogentisic acid; SONIA 2, suitability of nitisinone in alkaptonuria 2; sTYR, serum tyrosine; uHGA_24_, 24‐h urine homogentisic acid.

The percentage change against baseline (V0) was calculated for visits V12, V24, V36, and V48 in SONIA 2 and directly compared against corresponding values in the NAC namely, V1, V2, V3, and V4 (Table 3); these showed significantly greater lowering of uHGA_24_ by nitisinone for V12, V24, and V48 in the SONIA 2.

**TABLE 3:** Direct comparison of percentage change in uHGA24, sHGA, and sTYR at 12‐, 24‐, 36‐, and 48‐month visits in SONIA 2 and NAC—Mean (*SD*)

Measurements	Cohorts	12 month	24 month	36 month	48 month
uHGA_24_ (μmol/day)	SONIA 2	−99.5 (0.89)***	−98.3 (5.8) ***	−97.1 (13.0)	−97.7 (8.7)^a^
	NAC	−94.0 (4.9)	−93.7 (6.0)	−94.6 (3.8)	−94.3 (4.0)
sHGA (μmol/L)	SONIA 2	−97.5 (5.3)***	−92.7 (23.7)**	−89.4 (31.0)	−91.5 (21.2)^a^
	NAC	−80.6 (9.5)	80.2 (18.5)	−84.3 (10.2)	−80.9 (16.1)

*Note*: *p < 0.05; **p < 0.01; ***p < 0.001 for SONIA 2 versus NAC comparisons.

Abbreviations: NAC, National Alkaptonuria Centre; sHGA, serum homogentisic acid; SONIA 2, suitability of nitisinone in alkaptonuria 2; sTYR, serum tyrosine; uHGA_24_, 24‐h urine homogentisic acid.

### 3.3 Changes in sHGA at follow‐up in the NAC and SONIA 2

Changes in sHGA in the nitisinone and control groups in the NAC and SONIA 2 are shown in Figure S3A–D (Tables S3 and S4). Direct comparisons of baseline, 1‐, 2‐, 3‐, and 4‐year visits between SONIA 2 and NAC in the nitisinone group are shown in Table S2. sHGA was significantly lower at 1, 2, 3 and 4 years following nitisinone in SONIA 2 compared with NAC.

The percentage change against baseline (V0) was calculated for visits V12, V24, V36, and V48 in SONIA 2 and directly compared against corresponding values in the NAC namely, V1, V2, V3, and V4 (Table 3); these showed significantly greater lowering of sHGA by nitisinone for V12, V24, and V48 in SONIA 2.

### 3.4 Changes in sTYR at follow‐up in the NAC and SONIA 2

Changes in sTYR in the nitisinone and control groups in the NAC and SONIA 2 are shown in Figure S4A–D (Tables S3 and S4). Direct comparisons of baseline, 1‐, 2‐, 3‐, and 4‐year visits between SONIA 2 and NAC in the nitisinone group are shown in Table 2. sTYR was significantly higher at 1 and 2 years following nitisinone in SONIA 2 compared with NAC.

The percentage change against baseline (V0) was calculated for visits V12, V24, V36, and V48 in SONIA 2 and directly compared against corresponding values in the NAC namely, V1, V2, V3, and V4 (Table 3); these showed no significantly greater percentage increase in sTYR by nitisinone for V12, V24, V36, and V48 in SONIA 2.

### 3.5 Levels of sNIT at follow‐up in the NAC and SONIA 2

Levels of sNIT in the nitisinone groups in the NAC and SONIA 2 are shown in Figure S5A,B. Direct comparisons of the 1‐, 2‐, 3‐, and 4‐year visits between SONIA 2 and NAC in the nitisinone group are shown in Table 2. sNIT was significantly higher at all time points following nitisinone in SONIA 2 compared with NAC. Despite higher sNIT at later visits in SONIA 2, sHGA tended to increase over time (Figure S5C,D).

### 3.6 Slope and intersection of AKUSSI in the NAC and SONIA 2

**FIGURE 1:**
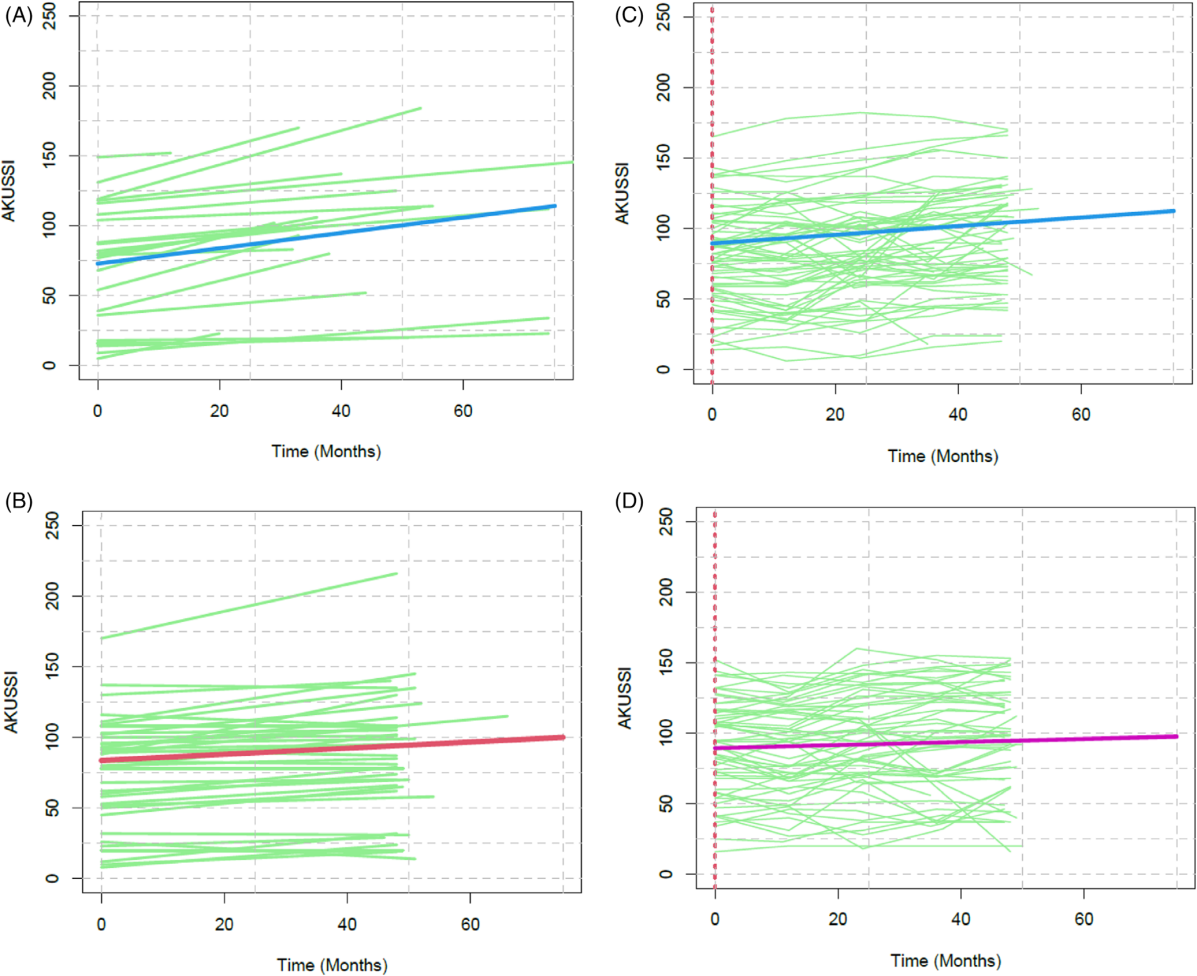
AKUSSI slopes in the NAC (A,B) and SONIA 2 (C,D) are shown. (A,B) In the NAC, AKUSSI scores rose on average at a rate of 0.55 every month in the control group, while it was at a rate of 0.19 every month in the nitisinone group. Please note that the control group showed varying intervals between the two visits, but the mean value was 44.3 months, with the nitisinone group being 48 months. (C,D) In the SONIA 2, AKUSSI scores rose on average at a rate of 0.30 every month in the control group, while it was at a rate of 0.06 every month in the nitisinone group. AKUSSI, Alkaptonuria Severity Score Index; NAC, National Alkaptonuria Centre; SONIA 2, suitability of nitisinone in alkaptonuria 2

In the NAC, AKUSSI scores rose on average at a rate of 0.55 per month in the control group, and extrapolating backwards to the *x*‐axis gives an intersection at an average time for an AKUSSI score equal to 0 at −132 months (minus 11 years). In the NAC nitisinone group, AKUSSI scores rose on average at a rate of 0.19 per month, and extrapolating backwards to the *x*‐axis gives an intersection at an average time for an AKUSSI score equal to 0 at −411 months (minus 34·25 years; Figure 1A,B; Table 4).

**TABLE 4:** The mean (*SD*) of the slopes and intersection on the *x*‐axis of the control and nitisinone groups in the NAC and SONIA 2 are shown

		NAC control	NAC nitisinone 2 mg	SONIA 2 control	SONIA 2 nitisinone 10 mg
Mean (*SD*) age at baseline (years)		47.4 (14.8)	46.8 (14.5)	47.7 (10.2)	49.0 (11.3)
Mean (*SD*) AKUSSI score at baseline		72.9 (42.3)	76.1 (37.8)	80.5 (33.4)	87.0 (34.2)
Slope AKUSSI score per month—mean (SEM)					
Unadjusted		0.55 (0.15)	0.19 (0.074)*	0.30 (0.06)	0.06 (0.065)*
Age		0 (0.006)	0 (0.003)	0.05 (0.12)	0.18 (0.126)
Sex		0 (0.192)	0.07 (0.099)	0 (0.006)	−0.01 (0.006)
X‐axis intersection—years (months)		−11.0 (−132)	−34.25 (−411)	−24.58 (−295)	−121.67 (−1460)
Disease onset in control or delayed to age using age of onset in control in nitisinone group (years)		36.4	70.65	23.12	144.79
Nitisinone‐induced delay compared to control (years)		23.25		97.09	

*Note*: The delay due to 2 mg and 10 mg nitisinone is shown. Data adjusted for age and sex are also shown. *NAC control versus NIT 2 mg *p* < 0.001 and SONIA 2 Control versus NIT 10 mg *p* < 0.001.

Abbreviations: AKUSSI, Alkaptonuria Severity Score Index; NAC, National Alkaptonuria Centre; NIT, nitisinone; SONIA 2, suitability of nitisinone in alkaptonuria 2.

In SONIA 2 control group, AKUSSI scores rose on average at a rate of 0.3 per month, and extrapolating backwards to the *x*‐axis gives an intersection at an average time for an AKUSSI score equal to 0 at minus 295 months (minus 24·58 years). In SONIA 2 nitisinone group, AKUSSI scores rose on average at a rate of 0·06 per month, and extrapolating backwards to the *x*‐axis gives an intersection at an average time for an AKUSSI score equal to 0 at −1460 months (minus 121·67 years; Figure 1C,D; Table 4).

All models were adjusted for patient age and sex (as centred variables). For no model was there any evidence of meaningful impact for either age or sex. Adjustment for age and sex in the NAC and SONIA 2 data sets had no notable impact on the slope analyses (Table 4).

### 3.7 Corneal keratopathy in the NAC and SONIA 2

In the NAC, there were three patients, out of a total of 60 patients treated with nitisinone, a frequency of 5%, with corneal keratopathy as shown in Table 5. All three were male. Patient 1 continued with the lower protein diet at 2 mg nitisinone daily. Patient 2 was on 2 mg nitisinone twice weekly and was poorly compliant with the lower protein diet. Patient 3 died from carcinoma of the stomach which came to light at the same time as the keratopathy.

**TABLE 5:** Corneal keratopathy in the NAC and SONIA 2

Patient no.	Sex	Age at onset (years)	Dose nitisinone (mg)	Onset keratopathy (months)	Slit‐lamp confirmation	sTYR μmol/L^a^
NAC						
1	M	25	2	36, B	Yes	964
2	M	21	2	1.5, U	Yes	941
3	M	55	2	3, B	No	NA
SONIA 2						
4	F	56	10	3, B	Yes	1022
5	F	69	10	14, U	No	1191
6	M	50	10	12, U	Yes	1236
7	M	39	10	6, B	Yes	1118
8	M	54	10	1, U	Yes	816
9	M	44	10	13, U	Yes	1036
10	M	29	10	36, B	Yes	1149
11	M	41	10	30, U	Yes	976
12	M	51	10	24, U	Yes	934
13	M	43	10	6, U	Yes	609

Abbreviations: B, Bilateral (following nitisinone in months); F, female; M, male; U, unilateral (following nitisinone in months).

^a^ Last serum tyrosine value before onset of keratopathy or withdrawal of nitisinone; serum tyrosine not always measured at diagnosis of keratopathy.

In the SONIA 2, there were 10 patients with keratopathy, with 9 confirmed by slit‐lamp examination, consisting of 2 women and 8 men (Table 5); one patient had clinical features of keratopathy unconfirmed by slit‐lamp examination due to not returning for this assessment. Of the patients with slit‐lamp confirmed keratopathy, resolution following discontinuation was only confirmed in eight patients. Five of the nine confirmed cases were bilateral corneal keratopathies. The age ranged from 29 to 69 years.

Overall, across SONIA 2 and the NAC, there were 11 men and 2 women who developed keratopathy. The numbers of patients with keratopathy in SONIA 2 at the Liverpool, Piešťany, and Paris sites were six, three, and one, respectively. The sTYR at the three sites in the nitisinone group was comparable, while the NAC values were lower (Table S5). In the keratopathy patients, most of the sTYR values were high despite such measurements not having been made immediately after the onset of keratopathy.

## 4 DISCUSSION

The current data analysis was carried out to assess the difference between the 2 mg and 10 mg daily doses of nitisinone used clinically in AKU. Prior publications suggest that data on disease progression, metabolic efficacy, and safety outcomes of both the nitisinone 2 mg daily and 10 mg daily are beneficial in AKU, with a greater HGA‐lowering effect at increasing doses.^11^, ^12^, ^14^, ^21^, ^22^ The nitisinone 10 mg dose has been approved by the European Medicines Agency for the treatment of adult patients with AKU. Nitisinone was initially developed for use in HT‐1, where the daily dose recommended is between 1 to 2 mg/kg body weight, with dietetic restriction of tyrosine and phenylalanine.^7^ To manage tyrosinaemia during nitisinone therapy, tyrosine and phenylalanine‐free amino acid supplements are widely used in addition to dietetic protein restriction; however, the efficacy of special amino acid supplements remains unproven as these are unpalatable. During the initial evaluation of nitisinone in AKU, doses such as 0.7 mg daily, 2.8 mg daily,^2^ 2.1mg,^9^ and 2 mg^10^ both as once‐ or twice‐daily administration were used. A formal dose–response of nitisinone in AKU carried out in SONIA 1 showed that an 8 mg dose was more efficacious than 2 mg in HGA‐lowering effect, with comparable effects on sTYR and short‐term safety.^14^ The commercially available 10‐mg capsule was then used in the SONIA 2 outcomes study.^12^ A later smaller study confirmed the metabolic superiority of nitisinone 8 mg compared to 2 mg in terms of decreasing the HGA, and like SONIA 1, also that serum tyrosine plateaued despite increasing nitisinone dose.^23^ One report described the use of 0.2 mg daily dose of nitisinone in a pregnant woman.^24^

**FIGURE 2:**
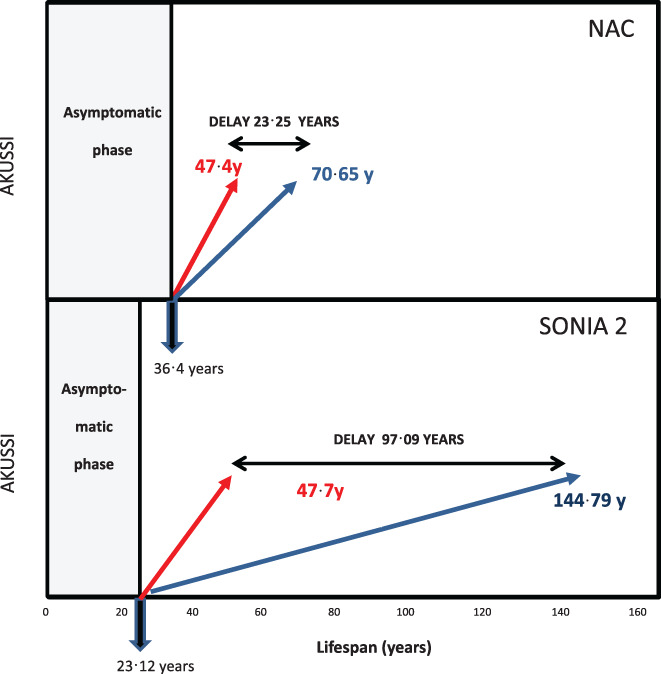
(A,B) Diagrammatic representation of (A) the effect of dose of nitisinone on AKU disease progression over the lifespan. The asymptomatic phase before the AKUSSI starts to increase is shown on the left side of the figure (grey box). Extrapolation of the control slope in NAC (red line) to time axis, in a cohort with an average age of 47.4 years, intersects the lifespan axis of this figure at 36.4 years, the point when the AKUSSI starts to increase. The slower slope in disease progression of 34.25 years in the NAC with nitisinone 2 mg (blue line) is shown from age 36.4 years, reaching baseline AKUSSI by age 70.65 years. This means a delay of 23.25 years following nitisinone 2 mg compared to control reaching baseline AKUSSI score. (B) Similarly, diagrammatic representation of the extrapolation of the control slope in SONIA 2 (red line) to time axis, in a cohort with an average age of 47.7 years, intersects results the lifespan axis of this figure at 24.58 years, the point when the AKUSSI starts to increase. The slower slope in disease progression of 121.67 years in the SONIA 2 with nitisinone 10 mg (blue line) is shown from age 24.58 years, reaching baseline AKUSSI by age 144.79 years. This means a delay of 97.09 years following nitisinone 10 mg compared to control reaching baseline AKUSSI score. AKU, Alkaptonuria; AKUSSI, AKU Severity Score Index; NAC, National Alkaptonuria Centre; SONIA 2, suitability of nitisinone in alkaptonuria 2

Progressive symptomatic AKU develops after the first two decades in life, as seen in Figure 2. The delay in AKU progression was studied in the NAC and SONIA 2. When one examines the NAC first, the untreated AKUSSI slope intersects the *x*‐axis (the time when it reaches zero) at minus 11 years. Since the baseline age in NAC was 47·4 years, the NAC intersect is at age 36.4 (47.4 − 11) years. In other words, AKUSSI increases from age 36.4 years to reach baseline values at 47.4 years. The NAC nitisinone 2 mg AKUSSI slope intersects the *x*‐axis at minus 34.25 years, a further 23.25 years delay. If nitisinone therapy was started at 36.4 years, when AKUSSI was zero, it would reach baseline AKUSSI values by age 70.65 (47.4 + 23.25) years, an overall delay of 23.25 (70.65 − 47·4) years.

In SONIA 2, the untreated AKUSSI slope intersects the *x*‐axis at minus 24.58 years. Since baseline age in SONIA 2 was 47.7 years, the AKUSSI slope intersect is at age 23.12 (47.7 − 24.58) years. In other words, AKUSSI increases from age 23.12 years to reach baseline values at 47.7 years. The nitisinone 10 mg AKUSSI slope intersects the *x*‐axis at minus 121.67 years. If nitisinone therapy was started at 23.12 years, it would reach baseline AKUSSI values by age 144.79 (23.12 + 121.67) years. The overall delay of AKU disease by 10 mg nitisinone is 97.09 (144.79 − 47.7) years. Disease progression is reduced by 23.25 and 97.09 years on nitisinone 2 mg and 10 mg, respectively, compared to no nitisinone.

Our direct comparisons between matched visits of SONIA 2 and NAC showed greater decreases following nitisinone in uHGA_24_ in SONIA 2 transiently. Unlike urine, sHGA has a causal role in the morbidity of AKU. In contrast to the transitory difference for uHGA_24_, the absolute values of sHGA showed significantly lower values following nitisinone in SONIA 2, and the percentage change was greater compared with the NAC, suggesting that 10 mg nitisinone had a greater HGA lowering effect than the 2 mg dose.

Nitisinone lowers HGA but increases circulating and tissue tyrosine levels.^25^ Direct comparison of sTYR between matched SONIA 2 and NAC visits showed lower levels in the NAC. There were also fewer keratopathies in the NAC, due to active management of the diet and possibly the lower dose. However, data demonstrate that even a dose as low as 1 mg daily increases sTYR above the target safety levels.^14^

The 10 mg nitisinone resulted in higher sNIT than the 2 mg dose. What has not been previously reported is the much higher sNIT values in the later visits of SONIA 2 only, but not in the NAC (Figure S5A–D). Nitisinone has been shown to inhibit cytochrome P450 mixed‐function oxidases and has been associated with drug interactions as a result^26^; we suggest that higher doses of nitisinone may inhibit its own catabolism over time leading to an increase in sNIT concentrations, as it is also catabolised by the cytochrome P450 mixed‐function oxidases.^27^ Despite this increase in sNIT later on in SONIA 2, paradoxically sHGA did not decrease further; nitisinone has been shown to inhibit organic anion transporters and putative mediators of renal HGA excretion and may account for this effect at the higher dose (Figure S5A–D).^28^

Nitisinone‐induced tyrosinaemia causes reversible dendritiform corneal keratopathy. In the NAC, there were three cases of keratopathy, a prevalence of 5%, all in the first 3 years after service commissioning, and it is likely that a dietetic management enabled effective management of the sTYR and mitigated the risk of keratopathy. In SONIA 2, there were 10 cases of keratopathy out of 69 nitisinone‐treated patients, a prevalence of 14·5%, where no formal dietetic input was used. Most of the cases of keratopathy exhibited sTYR greater than 900 μmol/L, consistent with previous data.^29^ Dietetic support following nitisinone in adult AKU patients habituated to consume protein unrestricted in adult AKU patients may minimise the occurrence of keratopathy.

Only two of the 13 patients with keratopathy were women, the reason being unclear. The single case of keratopathy reported in the NIH clinical trial was also male.^10^ Rats developed corneal keratopathy but not mice, and male rats have higher levels of sTYR compared to female rats following HPPD inhibitor administration, possibly due to sex hormone influence. We suggest that a similar mechanism may explain the male predisposition for keratopathy during nitisinone both in the SONIA 2 and the NAC.^30^, ^31^, ^32^

The data comparison carried out here has limitations. The SONIA 2 was a randomised trial while the NAC is a service. Nevertheless, the NAC data set collection was protocol‐driven and also overlapped temporally with SONIA 2. SONIA 2 was carried out in Liverpool, Piešťany, and Paris, while the NAC was only carried out in Liverpool. Similar AKUSSI assessments and outcomes have been collected in these two cohorts, but they were not identical. Diet was managed in the NAC, but not in SONIA 2, and may have influenced some outcomes especially regarding tyrosine and safety. The current analysis is important as it is unlikely that a direct comparison of nitisinone 2 mg and 10 mg on disease progression will be carried out in a clinical study.

In conclusion, nitisinone 10 mg leads to greater decreases in urine and serum HGA, due to greater HPPD inhibition, and greater increases in serum tyrosine, and more profoundly decreases disease progression. It is likely that a lower protein diet would improve safety following nitisinone at the 10 mg dose.

## CONFLICT OF INTERESTS

Nicolas Sireau received payments to the AKU Society to develop a health passport, a patient survey, a patient workshop, a video, a talk at a workshop, and a children's information booklet. Lakshminarayan R. Ranganath received fees for lectures and consultations from Swedish Orphan Biovitrum. Anna M Milan, Andrew T Hughes, Milad Khedr, Brendan P. Norman, MohammedAlsbou, Richard Imrich, Matthew Gornall, James A. Gallagher, and Richard Jackson declare that they have no conflict of interest.

## AUTHOR CONTRIBUTIONS

Lakshminarayan R. Ranganath, Nicolas Sireau, and James A. Gallagherpioneered the idea for SONIA 2, secured funding, and managed the study, drafting manuscript, and final approval of the manuscript. Lakshminarayan R. Ranganath and Milad Khedr are clinicians running the NAC service. Anna M. Milan, Andrew T Hughes, and Brendan P. Norman carried out the metabolic analyses, drafting manuscript, and final approval of the manuscript. Milad Khedr and Mohammed Alsbou assisted with conduct of the SONIA 2 study in Liverpool and Piešťany, respectively, drafting manuscript, and final approval of the manuscript. Assisted with conduct of the SONIA 2 study in Paris, drafting manuscript, and final approval of the manuscript. Richard Imrich assisted with conduct of study in Piešťany, drafting manuscript, and final approval of the manuscript. Matthew Gornall and Richard Jackson carried out all statistical aspects including the slope analysis, drafting manuscript, and final approval of the manuscript.

## ETHICS STATEMENT

All procedures followed were in accordance with the ethical standards of the responsible committee on human experimentation (institutional and national) and with the Helsinki Declaration of 1975, as revised in 2000 (5). Informed consent was obtained from all patients for being included in the study.

## Supporting information


**Table S1.** Serum tyrosine thresholds used for dietetic intervention in the NAC following nitisinone
**Table S2.** (A) AKU Severity Score Index (AKUSSI) shows the various features, how they were scored, in the NAC. (B) SONIA 2, and procedure is shown in italics
**Table S3.** SONIA 2 visit comparison within control and nitisinone groups (percentage change), for uHGA24, sHGA, sTYR, and AKUSSI
**Table S4.** NAC comparison between control and nitisinone visits (percentage change), for uHGA24, sHGA, sTYR, and AKUSSI
**Table S5.** Serum tyrosine at the three study sites in SONIA 2 and NAC (mean ± *SD*)
**Figure S1.** (A)The NAC study cohort and schedule of visits; only those who had completed 4 years of nitisinone therapy was used in the present analyses. The duration between the two visits of the control group varied, as can also be seen from Figure 1A. (B) the SONIA 2 study design; 69 patients received nitisinone while 69 controls did not. Following a baseline visit, patients visit study sites at Month 3 and then 12, 24, 36, and 48 months
**Figure S2.** (A–D) uHGA_24_ in the NAC (upper panels) and SONIA 2 (lower panels) is shown. In the NAC control group (S2A), a non‐statistical increase was seen at Month 48 (V0). In the NAC nitisinone group (S2B), values were 94.3%, 93.9%, 94.6%, and 94.2% lower at V1, V2, V3, and V4, compared to V0 (*p* < 0.001). In the SONIA 2 nitisinone group (S2D), values within group were 99.5%, 99.5%, 97.2%, 95.3%, and 95.6% lower at V3, V12, V24, V36, and V48, respectively, compared to V0 (*p* < .001)
**Figure S3.** (A–D) sHGA in the NAC (upper panels) and SONIA 2 (lower panels) is shown. In the NAC control group (S3A), a non‐statistical increase was seen at Month 48 (V0). In the NAC nitisinone group (S3B), values were 83.2%, 83.2%, 86.6%, and 85.1% lower at V1, V2, V3, and V4, compared to V0 (*p* < 0.001). In the SONIA 2 control group (S3C), there was a trend in the increase in sHGA in SONIA 2 when visits 36 and 48 months were compared with 3 months (*p* < 0.11). In the SONIA 2 nitisinone group (S3D), values within group were 97.7%, 97.7%, 93.1%, 91.7%, and 92.7% lower at V3, V12, V24, V36, and V48, respectively, compared to V0 (*p* < 0.001)
**Figure S4.** (A–D) sTYR in the NAC (upper panels) and SONIA 2 (lower panels) is shown. In the NAC control group (S4A), values were similar at V1 and V0. In the NAC nitisinone group (S4B), values were 12.1, 12, 13.8, and 13.5 times higher at V1, V2, V3, and V4, compared to V0 (*p* < 0.001). In the SONIA 2 nitisinone group (S4D), values within group were 13.6, 13.1, 12.4, 12.5, and 12.2 times lower at V3, V12, V24, V36, and V48, respectively, compared to V0 (*p* < 0.001)
**Figure S5.** (A–D) sNIT in the nitisinone groups of the NAC (upper left panel, S5A) and SONIA 2 (upper right panel, S5B) is shown. In SONIA 2 (S5B), values at V48 were increased compared to V3 (*p* < 0.01), and V12 (*p* < 0.05), indicating an increase over the 4 years. In SONIA 2, sNIT (lower left panel, S5C) and sHGA (lower right panel, S5D) without baseline values show trend to increase in sHGA in later visits despite increase in sNIT.

## Data Availability

SONIA 2 data access will be granted in response to qualified research requests. All de‐identified individual participant data, for patients with separate consent signed for this purpose, can be made available to researchers. Data will be shared based on the scientific merit of the proposal, that is, the proposal should be scientifically sound, ethical, and have the potential to contribute to the advancement of public health as well as the feasibility of the research proposal, that is, the requesting research team must be scientifically qualified and have the resources to conduct the proposed project. The data files would exclude data dictionaries that require user licences. Data could be made available following finalised regulatory authority review and end of any data exclusivity periods and ending after 36 months or until corresponding author is able to fulfil this obligation whichever is earlier. Further, the study protocol and statistical analysis plan can be made available. Proposals should be directed to j.a.gallagher@liverpool.ac.uk to gain access. Data requestors will need to sign a data access agreement. The data from the NAC can also similarly made available. Requests for data can be made to lrang@liv.ac.uk or milad.khedr@liverpoolft.nhs.uk.

## References

[1] O'Brien WM , La Du BN , Bunim JJ . Biochemical, pathologic and clinical aspects of alcaptonuria, ochronosis and ochronotic arthropathy: review of world literature (1584–1962). Am J Med. 1963;34:813‐838.

[2] Phornphutkul C , Introne WJ , Perry MB , et al. Natural history of alkaptonuria. N Engl J Med. 2002;347:2111‐2121. 12501223 10.1056/NEJMoa021736

[3] La Du BN , Zannoni VG , Laster L , Seegmiller JE . The nature of the defect in tyrosine metabolism in alcaptonuria. J Biol Chem. 1958;230:251‐260. 13502394

[4] Ranganath LR , Norman BP , Gallagher JA . Ochronotic pigmentation is caused by homogentisic acid and is the key event in alkaptonuria leading to the destructive consequences of the disease—A review. J Inherit Metab Dis. 2019;42:776‐792. 31282009 10.1002/jimd.12152

[5] Ranganath LR , Cox TF . Natural history of alkaptonuria revisited: analyses based on scoring systems. J Inherit Metab Dis. 2011;34:1141‐1151. 21748407 10.1007/s10545-011-9374-9

[6] Ranganath LR , Jarvis JC , Gallagher JA . Recent advances in management of alkaptonuria. J Clin Pathol. 2013;66:367‐373. 23486607 10.1136/jclinpath-2012-200877

[7] Lindstedt S , Holme E , Lock EA , et al. Treatment of hereditary tyrosinaemia type 1 by inhibition of 4‐hydroxyphenylpyruvate dioxygenase. Lancet. 1992;340:813‐817. 1383656 10.1016/0140-6736(92)92685-9

[8] Anikster Y , Nyhan WL , Gahl WA . NTBC and alkaptonuria. Am J Hum Genet. 1998;63:920‐921. 9718357 10.1086/302027 PMC1377415

[9] Suwannarat P , O'Brien K , Perry MB , et al. Use of nitisinone in patients with Alkaptonuria. Metabolism. 2005;54:719‐728. 15931605 10.1016/j.metabol.2004.12.017

[10] Introne WJ , Perry MB , Troendle J , et al. A 3‐year randomized therapeutic trial of nitisinone in Alkaptonuria. Mol Genet Metab. 2011;103:307‐314. 21620748 10.1016/j.ymgme.2011.04.016 PMC3148330

[11] Ranganath LR , Khedr M , Milan AM , et al. Nitisinone arrests ochronosis and decreases rate of progression of Alkaptonuria: evaluation of the effect of nitisinone in the United Kingdom national Alkaptonuria Centre. Mol Genet Metab. 2018;125:127‐134. 30055994 10.1016/j.ymgme.2018.07.011

[12] Ranganath LR , Psarelli EE , Arnoux JB , et al. Suitability of Nitisinone in Alkaptonuria 2 (SONIA 2) ‐ a randomised study on the efficacy and safety of nitisinone in alkaptonuria. Lancet Diabetes Endocrinol. 2020;8:762‐772. 32822600 10.1016/S2213-8587(20)30228-X

[13] Pharmacoeconomic Review Report: Nitisinone (Orfadin): (Sobi Canada Inc.) Indication: For the treatment of patients with hereditary tyrosinemia type 1 (HT‐1) in combination with dietary restriction of tyrosine and phenylalanine [Internet]. Ottawa (ON): Canadian Agency for Drugs and Technologies in Health; 2018. PMID: 30475548 (Checked 20th August 2021) 30475548

[14] Ranganath LR , Milan AM , Hughes AT , et al. Suitability of nitisinone in alkaptonuria 1 (SONIA 1): an international, multicentre, randomised, open‐label, control controlled, parallel‐group, dose–response study to investigate the effect of once daily nitisinone on 24‐h urinary homogentisic acid excretion in patients with alkaptonuria after 4 weeks of treatment. Ann Rheum Dis. 2016;75:362‐367. 25475116 10.1136/annrheumdis-2014-206033

[15] Griffin R , Psarelli EE , Cox TF , et al. Data on items of AKUSSI in Alkaptonuria collected over three years from the United Kingdom National Alkaptonuria Centre and the impact of nitisinone. Mol Genet Metab. 2018;20:1620‐1628. 10.1016/j.dib.2018.09.021 PMC6157456 30263914

[16] Häberle J . Suitability of nitisinone for alkaptonuria. Lancet Diabetes Endocrinol. 2020;8:732‐733. 32822593 10.1016/S2213-8587(20)30222-9

[17] Cox T , Ranganath L . A quantitative assessment of alkaptonuria: testing the reliability of two disease severity scoring systems. J Inherit Metab Dis. 2011;34:1153‐1162. 21744089 10.1007/s10545-011-9367-8

[18] Comparing Correlation Coefficients, Slopes, and Intercepts. file:///F:/USB2/USB2/AKU%20Further%20publications/AKU%20and%20nitisinone%20dose/2mg%20vs%2010%20mg/Review%20drafts/Final%20Version/Final/Slopes%20and%20intercepts.pdf (checked 20th August 2021)

[19] David Brown , Slope analysis – comparing placebo and treatment. https://www.ema.europa.eu/en/documents/presentation/presentation-disease-modifying-drug-development-statistical-design-analysis-david-brown_en.pdf

[20] Dyson AP , Tolooiyan A . Prediction and classification for finite element slope stability analysis by random field comparison. Comput. Geotech. 2019;109:117‐129.

[21] Ranganath LR , Khedr M , Vinjamuri S , Gallagher JA . Frequency, mechanism, diagnosis and treatment of ochronotic osteoporosis: the United Kingdom National Alkaptonuria Centre experience. Osteoporos Int. 2021;32:927‐938. 33118050 10.1007/s00198-020-05671-y

[22] Ranganath LR , Haseltine T , Khedr M , Fisher MF . Evaluating the aortic stenosis phenotype before and after the effect of homogentisic acid lowering therapy: analysis of a large cohort of eighty‐seven alkaptonuria patients. Mol Genet Metab. 2021;133(3):324‐331. 34059444 10.1016/j.ymgme.2021.05.007

[23] Gertsman I , Barshop BA , Panyard‐Davis J , Gangoiti JA , Nyhan WL . Metabolic effects of increasing doses of Nitisinone in the treatment of Alkaptonuria. JIMD Rep. 2015;24:13‐20. 25665838 10.1007/8904_2014_403 PMC4582031

[24] Sloboda N , Wiedemann A , Merten M , et al. Efficacy of low dose nitisinone in the management of alkaptonuria. Mol Genet Metab. 2019;127:184‐190. 31235217 10.1016/j.ymgme.2019.06.006

[25] Khedr M , Cooper M , Hughes AT , et al. Nitisinone causes acquired tyrosinosis in alkaptonuria. J Inherit Metab Dis. 2020;43:1014‐1023. 32083330 10.1002/jimd.12229

[26] Huledal G , Olsson B , Önnestam K , et al. Non randomized study on the potential of nitisinone to inhibit cytochrome P450 2C9, 2D6, 2E1 and the organic anion transporters OAT1 and OAT3 in healthy volunteers. Eur J Clin Pharmacol. 2019;75:313‐320. 30443705 10.1007/s00228-018-2581-7

[27] Summary of product characteristics (Annex 1). https://www.ema.europa.eu/en/documents/scientific-discussion/orfadin-epar-scientific-discussion_en.pdf (Checked 20th August 2021)

[28] Ranganath LR , Milan AM , Hughes AT , et al. Homogentisic acid is not only eliminated by glomerular filtration and tubular secretion but also produced in the kidney in alkaptonuria ‐ increase in circulating homogentisic acid in alkaptonuria with ageing is due to decreased renal clearance. J Inherit Metab Dis. 2020;43:737‐747. 31609457 10.1002/jimd.12181

[29] Lock EA , Ellis MK , Gaskin P , et al. From toxicological problem to therapeutic use: the discovery of the mode of action of 2‐(2‐nitro‐4‐trifluoromethylbenzoyl)‐1,3‐cyclohexanedione (NTBC), its toxicology and development as a drug. J Inherit Metab Dis. 1998;21:498‐506. 9728330 10.1023/a:1005458703363

[30] Nixon D , Herndon G , Wagner P . Mesotrione (ZA 1296). Tyrosine‐mediated toxicity in mice and rats. Submission to the United States Environmental Protection Agency. 2001. https://www3.epa.gov/pesticides/chem_search/cleared_reviews/csr_PC-122990_5-Jan-01_086.pdf (checked 20th August 2021)

[31] Low K & Tasheva M Mesotrione ‐ World Health Organization. http://www.fao.org/fileadmin/templates/agphome/documents/Pests_Pesticides/JMPR/Report2014/5.19_MESOTRIONE__277_.pdfmen (checked 20th August 2021)

[32] European Chemicals Agency Committee for Risk Assessment (2018). https://echa.europa.eu/documents/10162/5cc45ca7-fb5b-0fe8-198a-d3efa8c0d815 (checked 20th August 2021)

