# Supplementary material for: Comparing nitisinone 2 mg and 10 mg in the treatment of alkaptonuria—An approach using statistical modelling
Source: JIMD Rep. 2021 Nov 11;63(1):80–92. doi: 10.1002/jmd2.12261 (PMC8743340; doi:10.1002/jmd2.12261)
Supplement: Supplementary file 1 — Table S1. Serum tyrosine thresholds used for dietetic intervention in the NAC following nitisinone Table S2. (A) AKU Severity Score Index (AKUSSI) shows the various features, how they were scored, in the NAC. (B) SONIA 2, and procedure is shown in italics Table S3. SONIA 2 visit comparison within control and nitisinone groups (percentage change), for uHGA24, sHGA, sTYR, and AKUSSI Table S4. NAC comparison between control and nitisinone visits (percentage change), for uHGA24, sHGA, sTYR, and AKUSSI Table S5. Serum tyrosine at the three study sites in SONIA 2 and NAC (mean ± SD) Figure S1. (A)The NAC study cohort and schedule of visits; only those who had completed 4 years of nitisinone therapy was used in the present analyses. The duration between the two visits of the control group varied, as can also be seen from Figure 1A. (B) the SONIA 2 study design; 69 patients received nitisinone while 69 controls did not. Following a baseline visit, patients visit study sites at Month 3 and then 12, 24, 36, and 48 months Figure S2. (A–D) uHGA24 in the NAC (upper panels) and SONIA 2 (lower panels) is shown. In the NAC control group (S2A), a non‐statistical increase was seen at Month 48 (V0). In the NAC nitisinone group (S2B), values were 94.3%, 93.9%, 94.6%, and 94.2% lower at V1, V2, V3, and V4, compared to V0 (p < 0.001). In the SONIA 2 nitisinone group (S2D), values within group were 99.5%, 99.5%, 97.2%, 95.3%, and 95.6% lower at V3, V12, V24, V36, and V48, respectively, compared to V0 (p < .001) Figure S3. (A–D) sHGA in the NAC (upper panels) and SONIA 2 (lower panels) is shown. In the NAC control group (S3A), a non‐statistical increase was seen at Month 48 (V0). In the NAC nitisinone group (S3B), values were 83.2%, 83.2%, 86.6%, and 85.1% lower at V1, V2, V3, and V4, compared to V0 (p < 0.001). In the SONIA 2 control group (S3C), there was a trend in the increase in sHGA in SONIA 2 when visits 36 and 48 months were compared with 3 months (p < 0.11). In the SONIA 2 nitisin [file JMD2-63-80-s001.docx]

| Supplementary material | | |
| --- | --- | --- |
| Contents | | |
| Comparing nitisinone 2mg and 10mg in the treatment of alkaptonuria – an approach using statistical modelling | | |
|  | | Page No. |
| Table of contents | | 1 |
| 1.0 | Ethics issues in the NAC | 3 |
| 1.1 | Ethics approval | 3 |
| 2.0 | Study design of the NAC | 4 |
| 3.0 | Study design of SONIA 2 | 6 |
| 3.1 | Study design | 6 |
| 3.2 | Randomisation and masking | 7 |
| 3.3 | Procedures | 8 |
| 3.4 | Patients | 9 |
| 3.5 | Selection of study population | 10 |
| 3.6 | Treatment | 12 |
| 3.7 | Rescue from corneal keratopathy | 13 |
| 3.8 | Prior and concomitant therapy | 14 |
| 3.9. | Treatment compliance | 15 |
| 3.10 | Efficacy and safety assessments | 16 |
| 3.11 | Safety Assessment | 18 |
| 3.12 | Protocol amendments | 20 |
| 3.13 | Ethics committee approvals | 21 |
| 3.14 | Data handling procedures | 22 |
| 3.15 | Study oversight | 23 |
| 3.16 | DMC Oversight | 24 |
| 3.17 | Addressing bias in SONIA 2 | 25 |
| 4.0 | Legend to Supplementary tables and figures | 26 |
| 4.1 | Table S1a AKU Severity Score Index (AKUSSI) showing the various features, how they were scored, in the NAC | 28 |
| 4.1 | Table S1b SONIA 2, and procedure shown in italics*.* | 29 |
| 4.2 | Table S2. Serum tyrosine thresholds used for dietetic intervention in the NAC following nitisinone | 30 |
| 4.3 | Table S3. SONIA 2 visit comparison within control and nitisinone groups (% change), for uHGA24, sHGA, sTYR, AKUSSI. | 31 |
| 4.4 | Table S4. NAC comparison between control and nitisinone visits (% change), for uHGA24, sHGA, sTYR, AKUSSI. | 32 |
| 4.5 | Table S5. Serum tyrosine at the three study sites in SONIA 2 and NAC (Mean±SD). | 33 |
| 4.6 | Supplementary figures | 34 |
| 4.7 | Figure S1 (a, b) (a)The NAC study cohort and schedule of visits; Only those who had completed four years of nitisinone therapy was used in the present analyses. The duration between the two visits of the control group varied, as can also be seen from Figure 1a in the main manuscript.  (b) the SONIA 2 study design. 69 patients received nitisinone while 69 controls did not. Following a baseline visit, patients visits study sites at month 3 and then 12, 24, 36 and 48 months. | 34 |
| 4.8 | Figure S2 (a-d). uHGA_24_ in the NAC (upper panels) and SONIA 2 (lower panels) are shown.  In the NAC control group (S2a), a non-statistical increase was seen at month 48 (V0). In the NAC nitisinone group (S2b), values were 94·3, 93·9, 94·6 and 94·2% lower at V1, V2, V3 and V4, compared to V0 (p<0·001).  In the SONIA 2 nitisinone group (S2d), values within group were 99·5, 99·5, 97·2, 95·3 and 95·6% lower at V3, V12, V24, V36 and V48 respectively, compared to V0 (p<0·001). | 35 |
| 4.9 | Figure S3 (a-d). sHGA in the NAC (upper panels) and SONIA 2 (lower panels) are shown. All data are shown as boxplots with medians,  In the NAC control group (S3a), a non-statistical increase was seen at month 48 (V0). In the NAC nitisinone group (S3b), values were 83·2, 83·2, 86·6 and 85·1% lower at V1, V2, V3 and V4, compared to V0 (p<0·001).  In the SONIA 2 control group (S3c), there was a trend in increase in sHGA in SONIA 2 when visits 36 and 48 months were compared with 3 months (p<0.11).  In the SONIA 2 nitisinone group (S3d), values within group were 97·7, 97·7, 93·1, 91·7 and 92·7% lower at V3, V12, V24, V36 and V48 respectively, compared to V0 (p<0·001). | 36 |
| 4.10 | Figure S4 (a-d). sTYR in the NAC (upper panels) and SONIA 2 (lower panels) are shown. All data are shown as boxplots with medians,  In the NAC control group (S4a), values were similar at V-1 and V0. In the NAC nitisinone group (S4b), values were 12·1, 12, 13·8 and 13·5 times higher at V1, V2, V3 and V4, compared to V0 (p<0·001).  In the SONIA 2 nitisinone group (S4d), values within group were 13·6, 13·1, 12·4, 12·5 and 12·2 times lower at V3, V12, V24, V36 and V48 respectively, compared to V0 (p<0·001). | 37 |
| 4.11 | Figure S5 (a,b). sNIT in the nitisinone groups of the NAC (upper left panel, S5a) and SONIA 2 (upper right panel, S5b) are shown. All data are shown as boxplots with medians,  In the SONIA 2 nitisinone group (S5b), values at V48 were increased compared to V3 (p<0·01), and V12 (p<0·05), indicating an increase over the four years.  In SONIA 2, sNIT (lower left panel, S5c) and sHGA (lower right panel, S5d) without baseline values show trend to increase in sHGA in later visits despite increase in sNIT. | 38 |
|  | | |

1. **Ethics issues in the NAC**

**1.1. Ethics approval**

The Natural History Study was approved by NW REC: 07/Q1002/111, and this yielded data collected between 2007 and 2011; The rest of the data collected from the NAC was approved by the Institutional Audit Committee (Audit No:ACO3836), for annual audit.

Standard patient information sheets and signed consent was used in the research study between 2007 and 2011. Data from the NHS HSS approved service was collected and analysed as part of annual institutional audit (Audit no. ACO3836). A booklet describing all processes carried out in the NAC was given to each participant before they agreed to come to the NAC. Specific information was provided in the booklet that data collected would be published but subjects would not be identifiable from the dissemination process. Further, specific consent was obtained for the ear and eye photographs using the institutional consent mechanisms. Data from the NAC has been published in peer-reviewed journals previously along similar justifications. The highest standards of ethical and clinical practices were followed in delivering the service at the NAC. Our study complies with the Declaration of Helsinki, that the locally appointed ethics committee has approved the research protocol for the research aspects (NW REC: 07/Q1002/111), and that informed consent has been obtained from those subjects.

**2.0. Study design of the NAC**

All referred patients with confirmed AKU, through documented increase in urine homogentisic acid, attended the Royal Liverpool University Hospital between 2007 and 2020.

Attendance between 2007 and 2011, a single non-interventional visit (VR), was part of a research study (Natural History study in alkaptonuria; UK Research Ethics Committee Number 07/Q1002/111). No patient received nitisinone prior to 2012. Out of a total of 17 patients in this pre-2012 research study, ten also then attended the NAC when created in June 2012 at a baseline visit called V0. These ten patients are part of the NAC control group. Nitisinone 2mg was then administered to these ten patients and they form part of the 37 patients in the NAC nitisinone group.

The Highly Specialised Services, NHS England, commissioned and funded the NAC at the Royal Liverpool University Hospital in April 2012 to provide a service carrying out assessments, and offering treatments such as nitisinone. All referred patients, who completed four-years of follow-up with nitisinone, were included in the present dataset (the nitisinone group). The study protocol included a baseline visit (V0), followed by initiation of 2 mg oral nitisinone daily (administered off-license under approval from HSS) followed by up to four annual visits (V1, V2, V3, V4).

13 of those enrolled patients after 2012, had baseline and follow-up data collected as part of the study but were not eligible for nitisinone treatment per study requirements (as their residency was outside of the HSS approval region) – these 13 patients, along with the ten from the pre-2012 research study, formed the NAC control group of 23 patients.  The initial enrolment data for these 13 patients were included in the V(R) observational data, and their most recent follow-up data were recorded as time V0, thus capturing their untreated longitudinal data over a known time interval (Figure S1a).

Despite the NAC being a service to patients, systematic protocolised assessments agreed with the HSS were carried out at baseline and all subsequent visits (Table S2a). The assessments carried out in the NAC were similar to those carried out in the research study as described in detail previously, both in the NAC control and nitisinone groups.

Ear and eye ochronosis and spondyloarthropathy were assessed by systematic history taking by questionnaire and investigations. Eye and ear ochronosis were scored semi-quantitatively using standardised digital photographs taken in the Medical Photography department every visit. Alkaptonuria severity score index (AKUSSI) was derived (Table S2a). CT Bone densitometry was carried out to characterise the osteopenia. A questionnaire collected information about all treatment patients were taking at each visit; the questionnaire also elicited details of disease features such as pain in joints and spine annually. Technetium 99m-methyl diphosphonate (^99m^Tc MDP) scintigraphy was used in the natural history research study, but ^18^F-PETCT scintigraphy was used in the NAC. Table S2a lists all of the investigations used to score the AKUSSI. The transthoracic 2D echocardiogram assessments were performed using standard commercially available equipment and analysed with Philips software. All echocardiograms were performed by British Society of Echocardiography (BSE) accredited physiologists using the BSE minimum dataset. Aortic stenosis severity was defined mild, moderate or severe according to established national guidance.

A standard physical examination, including height and weight was carried out every visit. Serum and urine samples were collected from 2012. HGA was measured on acidiﬁed 24-h urine (u-HGA_24_) and fasting acidiﬁed serum samples from each visit as previously described by tandem mass spectrometry, were analysed for HGA, tyrosine and nitisinone (sHGA, sTYR, sNIT). Nitisinone 2mg oral daily was commenced on day three of the baseline visit, except for a smaller group of 13 patients (in the control group) who attended for assessment only. A full-time specialist dietician engaged with all patients on nitisinone to assess, monitor and manage their diet to minimise tyrosinaemia during nitisinone use to mitigate the unwanted effects of tyrosinaemia. Body weight, body composition and 7-day food diaries were used in assessment. Tyrosine was managed according to pragmatic thresholds shown in Table S2.

1. **Study design of SONIA 2**

# 3.1. Study design

SONIA 2 was a four-year, open-label, evaluator-blinded, multicentre, randomised, no-treatment controlled, parallel-group study. A formal interim analysis was planned when all patients completed 12 months of treatment. This analysis included the complete set of efficacy and safety data up to 12 months, thus including the final analysis of the primary endpoint. The purpose was to evaluate if data demonstrated results suitable for a regulatory application already at that stage, even though the study was to continue for another 3 years to collect more complete efficacy and safety data. The study design is summarised in Figure S2b.

The study was performed at three investigational sites: Liverpool (UK), Paris (France) and Piešťany (Slovakia). Independent Ethics Committee at each centre approved the study.

**3.2. Randomisation and masking**

Patients were randomly assigned to one of the two groups in a 1:1 ratio. The randomisation was stratified by study centre and age (≤ 55 years and > 55 years) and was carried out by using randomly permuted blocks (4 patients/block) within each study centre and age stratum. The study statistician created a program to randomly assign the patients to the two treatment groups using the SAS System. The randomisation was centrally implemented in the electronic CRF system (Viedoc®).

It is not possible to blind a study with nitisinone in AKU because one of the signs of the disease is that the urine darkens due to oxidation of excreted HGA. Patients can therefore easily notice if they are receiving active drug or not. Therefore, the control group received no placebo treatment. Instead, the study was evaluator-blinded as far as possible. Assessments which did not require direct contact between the evaluator and the patient (such as evaluation of images) were blinded during the entire study. The blinded evaluators were experts in their respective field, and never met the patients. Other assessments were made by objective measurements (Table S2b), such as that of bone density. It is, however, recognised, that reporting of subjective assessments may have introduced bias for some of the secondary endpoints, such as pain and quality-of-life assessments, and reporting of adverse events.

# 3.3. Procedures

In addition to a 24-h urine (u-HGA_24_) collected into acid for HGA and creatinine determination, fasting acidified serum for HGA, tyrosine and creatinine, a number of assessments and investigations were carried out (supplementary material). These included collection of medical history and physical examination, including those specific for AKU, a wide range of clinical outcome measures, including range of motion tests and quality of life assessments, safety assessment and other procedures shown in Table S2b.

*AKU Severity Score Index (AKUSSI) assessments*

The AKUSSI incorporates multiple, clinically meaningful AKU outcomes that can be described in a single score (Table S2b). All items included in the AKUSSI were assessed at baseline and yearly thereafter.

Patients visited study sites at 3 months, and then annually up to month 48; a close-out phone call took place at month 49. A questionnaire, completed by patients, collected safety information at 6, 18, 30 and 42 months.

At each visit, AEs and laboratory values were recorded. AEs included clinically significant signs and symptoms and abnormal test findings (e.g. laboratory analysis results, vital signs or ECG) that the investigator considered clinically significant and/or that led to a medical/surgical intervention including withdrawal of nitisinone or discontinuation from the study.

**3.4.** **Patients**

Patients with a well-documented AKU verified by increased urine HGA excretion and who were at least 25 years old and had any clinical manifestation in addition to increased HGA were eligible for inclusion in the study. Details of inclusion and exclusion criteria are described in this supplementary appendix. In all patients, diagnosis of AKU was also confirmed by *HGD* gene mutation identification performed during the study (data not shown). 140 AKU patients were to be randomised, equally distributed to two groups, to receive either nitisinone or no treatment (control). All patients provided written informed consent prior to inclusion.

**3.5.** **Selection of study population**

### *Inclusion criteria*

A patient needed to fulfil the following criteria in order to be included in the study:

1. Diagnosis of AKU.
2. Any clinical manifestations of AKU, such as clinical ochronosis or chronic back/joint pain.
3. Age ≥ 25 years.
4. Willing and able to visit the investigational site for study visits.
5. Signed written informed consent given.

### *Exclusion criteria*

The presence of any of the following excluded a patient from inclusion in the study:

1. Treatment with nitisinone within 3 months of randomisation.
2. Participation in another clinical study within 3 months of randomisation.
3. Known allergy to nitisinone or any of the constituents of the investigational product.
4. Female patient of child-bearing potential not using a reliable method of contraception.
5. Currently pregnant or lactating.
6. Current malignancy.
7. Uncontrolled hypertension (blood pressure greater than 180 mmHg systolic or greater than 95 mmHg diastolic).
8. Unstable cardiovascular disease.
9. Serum potassium < 3·0 mmol/L.
10. eGFR < 60 mL/min/1·73 m^2^.
11. ALT > 3 x upper limit of normal.
12. Haemoglobin < 10·0 g/dL.
13. Platelets < 100 x 10^9^/L.
14. Total white blood count < 3·0 x 10^9^/L or neutrophil count < 1·5 x 10^9^/L.
15. History of alcohol or drug abuse.
16. Psychiatric or somatic illness that interferes with compliance or communication with health care personnel.
17. Foreseeable inability to cooperate with given instructions or study procedures.
18. Any other medical condition which in the opinion of the investigator makes the patient unsuitable for inclusion.

### *Withdrawal of subjects from treatment or study*

A patient was withdrawn from treatment if the patient developed the following signs or symptoms which were judged by the investigator to be related to elevated tyrosine:

- Developed ocular signs or symptoms, or
- Developed a skin rash (including hyperkeratotic lesions)

Patients withdrawn due to an adverse event could not re-enter the study. Patients temporarily withdrawn from treatment due to suspected tyrosine toxicity could, however, continue in the study on a lower dose of nitisinone (2 mg) at the discretion of the investigator and only once all signs and symptoms of tyrosine toxicity had resolved. Patients who were temporarily withdrawn due to a suspected tyrosine toxicity, but not later confirmed, could continue in the study on the 10 mg dose. In both cases, dates for temporary withdrawal of nitisinone, the date when treatment is reinitiated, and the dose used after the pause, were recorded in the eCRF.

A patient was also to be withdrawn from the study if:

- Became pregnant.
- Developed allergy to nitisinone or any of the constituents of the investigational product.
- ALT increased to > 3 x upper limit of normal.
- Platelets decreased to < 100 x 10^9^/L.
- Total white blood count decreased to < 2·5 x 10^9^/L or neutrophils < 1·0 x 10^9^/L.

Furthermore, a patient was to be withdrawn from the study treatment if, in the opinion of the investigator, it was medically necessary, or if it was the wish of the patient.

**3.6.** **Treatment**

The dose used in SONIA 2 was 10 mg oral daily, based on the results of the SONIA 1 study, where daily urine HGA was decreased by 99·4 % at nitisinone 8 mg.^1^ In the short study of SONIA 1, a liquid nitisinone suspension was employed. For the much longer SONIA 2 study, 10 mg nitisinone (Orfadin®) capsules, closest to the 8 mg liquid suspension used in SONIA 1, were taken by patients first thing in the morning. The control group did not receive the study drug. A dose of 2 mg was used when a patient developed tyrosine-related adverse events, such as corneal keratopathy.

**3.7.** **Rescue from corneal keratopathy**

Nitisinone was withdrawn in patients who developed signs of ocular tyrosine-related adverse events (AEs). If feasible, once the symptoms had resolved (minimum 2 months after temporary withdrawal), nitisinone was reintroduced at a lower dose (2 mg daily). Alternatively, the patient was withdrawn from the study. If ocular tyrosine-related symptoms reappeared on the lower dose, nitisinone was permanently withdrawn and the patient was monitored until the symptoms resolved.

However, in the present slope analysis of the SONIA 2 data manuscript, the uHGA_24_, sHGA, sTYR and sNIT from these ‘rescued’ patients were used in the SONIA 2 nitisinone analysis unamended for the change in dose.

**3.8. Prior and concomitant therapy**

There were no restrictions regarding concomitant medications. Patients in both groups could freely use e.g. analgesics, anti-inflammatory drugs and others as needed to treat symptoms of AKU. Patients were allowed to continue on any chronic medication and any changes during the study were recorded, from the time of the screening and randomisation visit until the follow-up telephone call. Patients were not allowed to have used nitisinone within the 60 days prior to randomisation.

**3.9. Treatment compliance**

Product accountability records were kept by the pharmacy and investigator. All unused IMP was returned to the clinical study sites and measured. The amount consumed was compared to the expected consumption for the randomised dose.

**3.10. Efficacy and safety assessments**

Patients in the control arm underwent all the study procedures and investigations except they did not take nitisinone. All procedures were harmonised across all three study sites right at the beginning. All patients were to be evaluated with standard history taking and physical examination including those specific for AKU. For those assessments which were operator-dependent, the same persons were to conduct the test throughout the study, where possible. The persons performing these assessments were kept blinded as far as possible. Also, whenever possible, a central blinded assessor evaluated the results. Echocardiography, scintigraphic scans, X-rays, and photographs of eyes and ears were evaluated by completely blinded assessors.

A timed 24-h urine ((u-HGA_24_) was collected in bottles containing 30 mL of 5N H_2_SO_4_. Fasting blood samples were collected at each visit and an aliquot also acidified as previously described to stabilise the serum HGA.^1^ Urine was analysed for HGA, creatinine and urea. Creatinine was analysed by the Jaffe reaction (Roche Diagnostics, Germany). Serum analyses included HGA, tyrosine and nitisinone. The concentrations of tyrosine and HGA in serum and urine were measured by liquid chromatography tandem mass spectrometry. All analyses were performed on an Agilent 6490 Triple Quadrupole mass spectrometer with Jet-Stream electrospray ionisation coupled with an Agilent 1290 inﬁnity II Ultra High-Performance Liquid Chromatography (UHPLC) pump and HTC autosampler. All serum and urine quantitation analyses were performed by the Department of Clinical Biochemistry and Metabolic Medicine at the Royal Liverpool University Hospital as described previously.

Photographs of the eyes and ears were carried out at each visit. Pigmentation of eyes and ears was assessed using clinical photographs. Photographs were taken using standardised conditions as far as possible. The photographs included pictures of each eye (temporal and nasal aspects) separately, and of each ear. Plain radiographs, anteroposterior and lateral views X-rays, including the whole spine and pelvis (from Cobb angle measurements for kyphosis and scoliosis), were performed. Standard abdominal ultrasonography was used to assess renal and prostate stones; in addition, patients were asked to record details about possible episodes of renal or prostate stones between visits in a diary. Bone densitometry using Dual Energy X-Ray Absorptiometry was measured at one hip, usually the non-dominant one. A transthoracic echocardiogram assessed cardiac function and extent of valvular disease annually. Transthoracic echocardiography was used to assess aortic stenosis and sclerosis. The echocardiograms were performed according to British Society of Echocardiography Guidelines for Valve Quantification. The echocardiograms were evaluated by a central blinded assessor. In addition to the categorical stratifications of aortic valve involvement, both the peak aortic velocity and the aortic valve area, calculated by the continuity equation method were employed for assessment. Audiometry was used to assess hearing loss; both ears were tested through a range of frequencies (0.5, 1, 2, 3, 4, 6 and 8 kHz) and hearing loss (dB) recorded for each frequency, via a series of sound exposures. All fractures and ruptures were to be reported by the patient. At the baseline visit, the patient was asked about any previous adult fractures and ruptures. New fractures and ruptures occurring after study start were reported by the patient in a diary.

Musculoskeletal system was evaluated for symptoms (pain) in 14 joint areas (hips, knees, ankles, feet, shoulders, elbows and hands) and 4 spine areas (cervical, thoracic, lumber and sacroiliac); a score of 1 was given for presence of pain in the joint areas within the previous week and a score of 2 was given for pain in spine areas similarly. A scintigraphic scan was performed at the initial visit and each subsequent visit; this was a ^18F^PETCT at the Liverpool site and Technetium 99m-methyl diphosphonate at the Piešťany and Paris sites; joint and spine areas were scored for involvement. A score of 2 each was given for presence of tracer uptake in the joint areas already described and a score of 4 each was given for pain in spine within the previous week (cervical, thoracic, lumber and sacroiliac), and associated areas (costochondral and pubic symphysis) similarly.

Full ocular examinations including slit lamp examination and corneal photographs were completed at each visit. Otoscopic examination was carried out with photographs of tympanic membrane performed. Advice on limiting protein intake was provided and reinforced by a diet sheet; a dietician was not involved since the previous randomised study did not reveal significant corneal toxicity.

**3.11. Safety Assessment**

At each visit, adverse events (AEs) and laboratory values were recorded. Routine laboratory processes at each clinical study site were employed to measure biochemistry and haematology profiles. AEs included abnormal test findings (e.g. laboratory analysis results, vital signs or ECG) that the investigator considered clinically significant and/or that led to a medical/surgical intervention including withdrawal of IMPs or discontinuation from the study. If the test was associated with accompanying symptoms, the symptom, not the test result, was recorded as an AE. All directly observed AEs, and all AEs spontaneously reported by the patient, were recorded in the eCRF. The question asked was “Have you had any health problems since your last clinic visit?”.

Tyrosine can occasionally cause idiosyncratic reversible corneal dendritiform keratopathy and skin rash. Elevated tyrosine may lead to ocular signs and symptoms, including corneal ulcers, corneal opacities, keratitis, conjunctivitis, eye pain, and photophobia.

At the start of the study, all patients were provided with instructions to contact the investigator and visit the investigational site as soon as possible if they developed a skin rash or photophobia, eye pain, or signs of inflammation such as redness, swelling, or burning of the eyes during the study. In case a patient developed such signs or symptoms, this was reported and followed up as an AE.

Nitisinone was withdrawn in patients who developed signs of tyrosine-related AEs. If feasible, once the symptoms had resolved (minimum 2 months after temporary withdrawal), nitisinone was reintroduced at a lower dose (2 mg/day). Alternatively, the patient was withdrawn from the study. If ocular tyrosine-related symptoms reappeared on the lower dose, nitisinone was permanently withdrawn and the patient was to be monitored until the symptoms resolved. Nitisinone was not reintroduced again. Patients who were temporarily withdrawn due to a suspected, but not later confirmed, tyrosine-related AE, continued in the study on the 10mg dose.

Other safety monitoring included laboratory tests (clinical chemistry and haematology), electrocardiogram, and vital signs (blood pressure, pulse, temperature). Patients completed a safety monitoring questionnaire between the scheduled site visits, at months 6, 18, 30 and 42 in order to collect safety information specifically concerning possible tyrosine-related AEs. The patients were asked if they had had any problems with their eyes or skin since their last visit to the study site.

Patients were asked to record information about any emerging AKU-related events in a diary (event and date, treating physician’s name and institution) on the following items as they occurred, namely fractures, muscle/tendon/ligament ruptures, renal stones, prostate stones, arthroscopies, and joint replacements.

A corneal eye examination was performed at all scheduled visits to the clinic. This consisted of corneal photographs with or without slit-lamp examination.

A total of nine patients following detection of corneal keratopathy, discontinued the 10mg dose, and switched to the rescue plan nitisinone dose of 2mg after a recovery period of two months. However, in the present slope analysis of the SONIA 2 data manuscript, the uHGA_24_, sHGA, sTYR and sNIT from these ‘rescued’ patients were used in the SONIA 2 nitisinone analysis unamended for the change in dose.

**3.12. Protocol amendments**

The amendments mostly contained corrections and clarifications. There were no major changes that affected the performance of the study in any important way. Amendment 1 added results from SONIA 1 in the background and defined the dose. This information was missing when Version 1 was written. Amendment 2 only contained corrections and clarifications. In amendment 3, MRI was reclassified from a secondary to an exploratory assessment and changes to the study staff were presented, along with further clarifications.

**3.13. Ethics committee approvals**

The reference numbers for the ethics approvals are as follows:

EC Liverpool (NRES Committee North-West – Liverpool Central) Reference number: 13/NW/0567.

EC Piešťany (NURCH Ethica Committee, National Institute of Rheumatic Diseases, Ivana Krasku 4,92101 Piešťany, Slovak Republic) Reference number: 04196/0029/001/001.

EC Paris (EC Ile De France II, hospital Necker 149 Rue de Sevres 75 743 Paris Cedex 15, Porte N2, 1er etage, France). Reference number: 2013-08-08.

**3.14. Data handling procedures**

The data for the SONIA 2 trial has been collected using an electronic Case Report Form (eCRF). The eCRF is developed in Viedoc 3, a 21 CFR Part 11 compliant web-based software, based on the study protocol. The set-up was approved after User Acceptance Testing by Sponsor and CRO. Data was entered by the Principal investigators or designees. The completed data was checked on site with the source for completeness and accuracy. Central assessors, experts on specific examinations, reviewed specific tests and provided the results. These results were also captured in the eCRF. Principal investigators confirmed all data to be complete and correct by signing off all completed data.

Medical History terms and Adverse Events were reported in the eCRF and coded with the MSSO MedDRA dictionary version 21.1. Concomitant medication was coded with the ATC codes from the WHO drug dictionary ATC/DDD Index 2019. All coding was approved by a Medical Doctor and Pharmacist.

Data exports were provided to the Trial Statistician from the University of Liverpool in SAS format. Once the data extract was received by the Trial statistician, then any data handling and analysis was performed in SAS and datasets were stored in a secure University of Liverpool server. *In the present manuscript, the slope analysis was carried out post-hoc by the statistician.*

**3.15. Study oversight**

The study was conducted at three sites, Liverpool (United Kingdom), Paris (France) and Piešťany (Slovakia) from May 2014 to February 2019. Data were recorded by investigators at each site, collected, and monitored by the Contract Research Organisation PSR Group (Amsterdam, Netherlands). The protocol and amendments were approved by the relevant ethics review boards and national regulatory authorities. Written informed consent was obtained from all patients before any study procedures. An independent Data and Safety Monitoring Board was assigned to evaluate the safety data.

**3.16. DMC Oversight**

DMC meetings were held once yearly. The members of the DMC were Professor RJ Moots (Chair), Dr Andy Vail (Independent Statistician), Dr Theresa Barnes (Rheumatologist) and Dr Patrick McKiernan (Metabolic Physician). No DMC review resulted in a protocol amendment. The remit of the DMC was to consider only safety as the intention was always to complete the 4-year duration if safety was acceptable regardless of efficacy data at 12 months. The DMC charter reflected this decision.

**3.17. Addressing bias in SONIA 2**

Randomisation bias excluded as far as possible by rigorous attention to masking and concealment. We could not blind patients as already explained and therefore there is the possibility of bias due to patient expectations especially in relation to pain scores, quality of life questionnaires and possible active range of motion. Drop-outs were not followed up and it is unknown if any bias was introduced. Intention-to-treat analysis was followed as described a priori in statistical analysis plan. All data collected in drop-outs were analysed. Despite involvement of industry partners, every care was taken to minimise the effect of competing interests. All data including positive and negative results has been declared to minimise publication bias.

1. **Legend to Supplementary tables and figures**

Table S1 a, b. (a) AKU Severity Score Index (AKUSSI) showing the various features, how they were scored, in the NAC and (b) SONIA 2, and procedure shown in italics*.*

Table S2. Serum tyrosine thresholds used for dietetic intervention in the NAC following nitisinone

Table S3. SONIA 2 visit comparison within control and nitisinone groups (% change), for uHGA24, sHGA, sTYR, AKUSSI.

Table S4. NAC comparison between control and nitisinone visits (% change), for uHGA24, sHGA, sTYR, AKUSSI.

Table S5. Serum tyrosine at the three study sites in SONIA 2 and NAC (Mean±SD).

Figure S1 (a, b). (a)The NAC study cohort and schedule of visits; Only those who had completed four years of nitisinone therapy was used in the present analyses. The duration between the two visits of the control group varied, as can also be seen from Figure 1a. (b) the SONIA 2 study design. 69 patients received nitisinone while 69 controls did not. Following a baseline visit, patients visits study sites at month 3 and then 12, 24, 36 and 48 months.

Figure S2 (a-d). uHGA_24_ in the NAC (upper panels) and SONIA 2 (lower panels) are shown. In the NAC control group (S2a), a non-statistical increase was seen at month 48 (V0). In the NAC nitisinone group (S2b), values were 94·3, 93·9, 94·6 and 94·2% lower at V1, V2, V3 and V4, compared to V0 (p<0·001). In the SONIA 2 nitisinone group (S2d), values within group were 99·5, 99·5, 97·2, 95·3 and 95·6% lower at V3, V12, V24, V36 and V48 respectively, compared to V0 (p<0·001).

Figure S3 (a-d). sHGA in the NAC (upper panels) and SONIA 2 (lower panels) are shown. In the NAC control group (S3a), a non-statistical increase was seen at month 48 (V0). In the NAC nitisinone group (S3b), values were 83·2, 83·2, 86·6 and 85·1% lower at V1, V2, V3 and V4, compared to V0 (p<0·001). In the SONIA 2 control group (S3c), there was a trend in increase in sHGA in SONIA 2 when visits 36 and 48 months were compared with 3 months (p<0.11). In the SONIA 2 nitisinone group (S3d), values within group were 97·7, 97·7, 93·1, 91·7 and 92·7% lower at V3, V12, V24, V36 and V48 respectively, compared to V0 (p<0·001).

Figure S4 (a-d). sTYR in the NAC (upper panels) and SONIA 2 (lower panels) are shown. In the NAC control group (S4a), values were similar at V-1 and V0. In the NAC nitisinone group (S4b), values were 12·1, 12, 13·8 and 13·5 times higher at V1, V2, V3 and V4, compared to V0 (p<0·001). In the SONIA 2 nitisinone group (S4d), values within group were 13·6, 13·1, 12·4, 12·5 and 12·2 times lower at V3, V12, V24, V36 and V48 respectively, compared to V0 (p<0·001).

Figure S5 (a-d). sNIT in the nitisinone groups of the NAC (upper left panel, S5a) and SONIA 2 (upper right panel, S5b) are shown. In SONIA 2 (S5b), values at V48 were increased compared to V3 (p<0·01), and V12 (p<0·05), indicating an increase over the four years. In SONIA 2, sNIT (lower left panel, S5c) and sHGA (lower right panel, S5d) without baseline values show trend to increase in sHGA in later visits despite increase in sNIT.

**4.1.** Table S1.

| Table S1a. AKU Severity Score Index (AKUSSI) used in the NAC showing the various features, the scoring, and *procedure shown in italics* | | | | | | | | | |
| --- | --- | --- | --- | --- | --- | --- | --- | --- | --- |
| Feature | | | Score | Feature | | | | | Score |
| CLINICAL FEATURES (excluding spine and joint) | | | | | | | | | |
| Eye pigment *(Standardised Medical Photography)* | | | | | | | | | |
| Right eye (Nasal)* | | | | Left eye (Nasal)* | | | | | |
| Right eye (Temporal)* | | | | Left eye (Temporal)* | | | | | |
| Ear pigment *(Standardised Medical Photography)* | | | | | | | | | |
| Right ear | Slight | | 2 | Left ear | Slight | | | | 2 |
|  | Marked | | 4 |  | Marked | | | | 4 |
| Stones *(Ultrasonography of abdomen and pelvis)* | | | | | | | | | |
| Prostate Stones | | Per episode | 4 | Renal Stones | Per episode | | | | 4 |
| Bone mineral density of hip  *CT BMD* | | Grade (T-scores)  ≥ -1·0  -1·0 | 0  4 | Hearing impairment *(Questionnaire)* | | | | 4 | |
| Adult fracture *(Questionnaire)* | | Per fracture | 8 | Ligament rupture | | Per rupture | | | 8 |
| Tendon rupture *(Questionnaire)* | | Per rupture | 8 | Muscle rupture | | Per rupture | | | 8 |
| Heart *(Transthoracic echocardiography)* | | | | | | | | | |
| Normal  Aortic sclerosis | | | 0  6 | Aortic valve stenosis | | | Mild  Moderate  Severe | | 8  10  12 |
| JOINT FEATURES | | | | | | | | |  |
| Clinical joint pain (1 for each large joint area; hips, knees, ankles, feet, shoulders, elbows, wrists & hands - right and left sides = 14 joint areas) *(Questionnaire)* | | | | | | | | | Max 14 |
| Non-spine joint disease (2 for each large joint area; hips, knees, ankles, feet, shoulders, elbows, wrists & hands - right and left sides = 14 joint areas) *(either* Technetium*99m-methyl diphosphonate or ^18F^PETCT)* | | | | | | | | | Max 28 |
| Arthroscopy (*Questionnaire*) | | | | | | | | | 2 each |
| Arthroplasty (*Questionnaire*) | | | | | | | | | 4 each |
| SPINE FEATURES | | | | | | | | |  |
| Clinical spinal pain (2 each for cervical, thoracic, lumbar, sacroiliac) *(Questionnaire)* | | | | | | | | | Max 8 |
| Spine disease (4 each for pubic symphysis, ribs, sacroiliac, lumbar, thoracic, cervical)  *(either* Technetium*99m-methyl diphosphonate or ^18F^PETCT)* | | | | | | | | | Max 24 |
| Ochronosis scores for eyes and ears + Joint pain and PETCT scores + Spine pain and PETCT scores = AKUSSI | | | | | | | | |  |
| *NAC scoring: Eye pigmentation: 1, 2 and 3 points for slight, moderate and marked conjunctival pigmentation and 4, 6 and 8 points for slight, moderate and marked scleral pigmentation  Sum of eye pigment right eye + left eye (nasal + temporal in each eye) + right ear pigment + left ear pigment = ochronosis score  Joint Pain was scored 1 or 0 points for yes or no response for the 14 joint areas to the question – ‘During the last week, have you  experienced any pain in joint X?’  Spine Pain was scored 1 or 0 points for yes or no response for the 4 spine areas to the question – ‘During the last week, have you  experienced any pain in spine area X?’  Joint pain + Joint PETCT = JOINT score  Spine pain + Spine PETCT (plus pubic symphysis and costochondral) = SPINE score | | | | | | | | | |

| Table S1 b. AKU Severity Score Index (AKUSSI) in the SONIA 2 (*procedure shown in italics*) | | | | | | | | | | | | |
| --- | --- | --- | --- | --- | --- | --- | --- | --- | --- | --- | --- | --- |
| Feature | | | | | Score | Feature | | | | | | Score |
| CLINICAL FEATURES (excluding spine and joint) | | | | | | | | | | | | |
| Eye pigment *(Standardised Medical Photography)* | | | | | | | | | | | | |
| Right eye (Nasal) | | | Slight | | 4 | Left eye (Nasal) | Slight | | | | | 4 |
|  | | | Marked | | 8 |  | Marked | | | | | 8 |
| Right eye (Temporal) | | | Slight | | 4 | Left eye (Temporal) | Slight | | | | | 4 |
|  | | | Marked | | 8 |  | Marked | | | | | 8 |
| Ear pigment *(Standardised Medical Photography)* | | | | | | | | | | | | |
| Right ear | | Slight | | | 2 | Left ear | Slight | | | | | 2 |
|  | | Marked | | | 4 |  | Marked | | | | | 4 |
| Stones *(Ultrasonography of abdomen and pelvis)* | | | | | | | | | | | | |
| Prostate Stones | | | Per episode | | 4 | Renal Stones | Per episode | | | | | 4 |
| Musculoskeletal | | | | | | | | | | | | |
| Bone mineral density of hip  *Dual Energy X-Ray Absorbtiometry (DEXA)* | | | Grade (T-scores)  ≥ -1·0  -1·0 to -1·7  -1·8 to -2·4  < -2·5 | | 0  2  4  6 |  | | | | | | |
| Adult fracture  *(Questionnaire)* | | | Per fracture | | 8 | Ligament rupture | | Per rupture | | | | 8 |
| Tendon rupture  *(Questionnaire)* | | | Per rupture | | 8 | Muscle rupture | | Per rupture | | | | 8 |
| Heart *(Transthoracic echocardiography)* | | | | | | | | | | | | |
| Normal  Aortic sclerosis | | | | | 0  4 | Aortic valve stenosis | | | Mild  Moderate  Severe | | | 8  10  12 |
| ENT *(Audiometry)* | | | | | | | | | | | | |
| Hearing impairment  Graded on audiometry (dB loss), per ear | ≤ 20  21-35 (mild)  36-60 (moderate)  >60 (severe) | | | | 0  1  2  4 | Dark tympanic membrane  *(Otoscopic examination)* | | | | Per ear | | 6 |
| JOINT FEATURES | | | | | | | | | | | |  |
| Clinical joint pain (1 for each large joint area; hips, knees, ankles, feet, shoulders, elbows, wrists & hands - right and left sides = 14 joint areas) *(Questionnaire)* | | | | | | | | | | | | Max 14 |
| Non-spine joint disease (2 for each large joint area; hips, knees, ankles, feet, shoulders, elbows, wrists & hands - right and left sides = 14 joint areas) *(either* Technetium*99m-methyl diphosphonate or ^18F^PETCT)* | | | | | | | | | | | | Max 28 |
| Arthroscopies *(Questionnaire)* | | | | | | | | | | | | 2 each |
| Joint replacements *(Questionnaire)* | | | | | | | | | | | | 4 each |
| SPINE FEATURES | | | | | | | | | | | |  |
| Clinical spinal pain (2 each for cervical, thoracic, lumbar, sacroiliac) *(Questionnaire)* | | | | | | | | | | | | Max 8 |
| Spine disease (4 each for pubic symphysis, ribs, sacroiliac, lumbar, thoracic, cervical)  *(either* Technetium*99m-methyl diphosphonate or ^18F^PETCT)* | | | | | | | | | | | | Max 24 |
| Kyphosis  *(X-Ray Lateral Spine and pelvis)*  (Cobb angles) | | | | <45  45^.^-60  >60 | 0  3  6 | Scoliosis  *X-Ray antero-posterior Spine and pelvis*  (Cobb angles) | | | | | <5  5-20  21-30  >30 | 0  2  4  6 |

**4.2.** Table S2.

| Table S2. Serum tyrosine thresholds used for dietetic intervention in the NAC following nitisinone | |
| --- | --- |
| sTYR (µmol/L) | Action |
| <500 | Acceptable, no further action |
| 501 – 700 | Institute 0·9g/kg protein in diet |
| 701 – 900 | Institute 0·8g/kg protein in diet |
| >900 | Institute 0·8g/kg protein in diet, plus tyrosine-free amino-acid supplements |
| Keratopathy | Discontinue nitisinone and restart after 2months, intensify dietary protein restriction |

**4.3.** Table S3.

| Table S3. SONIA 2 Visit comparison within control and nitisinone groups against baseline (% change) | | | | | | | | | | | | | |
| --- | --- | --- | --- | --- | --- | --- | --- | --- | --- | --- | --- | --- | --- |
|  | Within control group comparison against baseline | | | | | | Within nitisinone group comparison against baseline | | | | | | |
|  | V0 | V3 | V12 | V24 | V36 | V48 | V0 | V3 | V12 | V24 | V36 | V48 |  |
| uHGA24 |  | -10·5 | -16·8 | -16·7 | -8·4 | -6·1 |  | -99·5* | -99·5* | -97·2* | -95·3* | -95·6* |  |
| sHGA |  | 0 | 2·1 | 14·1 | 41 | 32·5 |  | -97·7* | -97·7* | -93·1* | -91·7* | -92·7* |  |
| sTYR |  | 36·8 | 36·8 | -5·3 | -5·3 | 15·8 |  | 1356* | 1306* | 1235* | 1248* | 1217* |  |
| AKUSSI |  |  | -1·5 | 3·2 | 12·1 | 19 |  |  | -3·1 | 4·8 | 2·2 | 9·1 |  |
|  | | | | | | | | | | | | | |
| SONIA 2 Visit comparison between control and nitisinone groups (% change) | | | | | | | | | | | | | |
|  | V0 | | V3 | | V12 | | V24 | | V36 | | V48 | |  |
| uHGA24 | -1·1 | | -99·5* | | -99·4* | | -96·6* | | -94·9* | | -95·4* | |  |
| sHGA | 7·1 | | -97·5* | | -97·6* | | -93·5* | | -93·7* | | -94·1* | |  |
| sTYR | 1·2 | | 1382* | | 1369* | | 1242* | | 1210* | | 1246* | |  |
| AKUSSI | 8·08 | |  | | 6·31 | | 9·76 | | -1·44 | | -0·94 | |  |
| *p<0.001 | | | | | | | | | | | | | |

**4.4.** Table S4.

| Table S4. NAC comparison between control and nitisinone visits (% change) | | | | | |
| --- | --- | --- | --- | --- | --- |
|  | V-1^≠^ | V1 | V2 | V3 | V4 |
| uHGA24 | 17·1 | -94·3* | -93·9* | -94·6* | -94·2* |
| sHGA | 29·1 | -83·2* | -83·2* | -86·6* | -85·1* |
| sTYR | 22·6 | 1212* | 1201* | 1382* | 1351* |
| AKUSSI | 31·3* | 2·4 | 9·1 | 9·7 | 14·2 |
| V-1^≠^ comparisons were made against V0≠ (n=23); V1 to V4 comparisons were made against V0 (n=37)  *p<0.001 | | | | | |

**4.5**. Table S5.

| Table S5. Serum tyrosine (µmol/L) at the three study sites in SONIA 2 and NAC [Mean (SD)]. | | | | |
| --- | --- | --- | --- | --- |
|  | Liverpool | Piešťany | Paris | NAC |
| V3 | 934 (319) | 950 (180) | 905 (262) |  |
| V12 | 989 (248) | 920 (170) | 829 (188) | 707 (153) |
| V24 | 842 (260) | 840 (243) | 968 (215) | 701 (210) |
| V36 | 902 (293) | 778 (306) | 1037 (244) | 799 (156) |
| V48 | 923 (409) | 752 (239) | 986 (303) | 782 (181) |

| Figure S1a. The NAC study cohort and visits to the NAC | | | | | |
| --- | --- | --- | --- | --- | --- |
| VR | V0 | V1 | V2 | V3 | V4 |
| Pre-2012 *^¥^  n=10 |  |  |  |  |  |
| Post-2012 *  n=13 |  |  |  |  |  |
| NAC NIT GROUP  N=37 |  |  |  |  |  |
|  | *Represents 44.3 months between VR and V0 visits; did not receive nitisinone  ^¥^At V0, went on to receive nitisinone 2mg daily until V4; data contained in NAC nitisinone group (n = 37) | | | | |

**4.6.** Supplementary figures

**4.7.** Figure S1 (a, b).


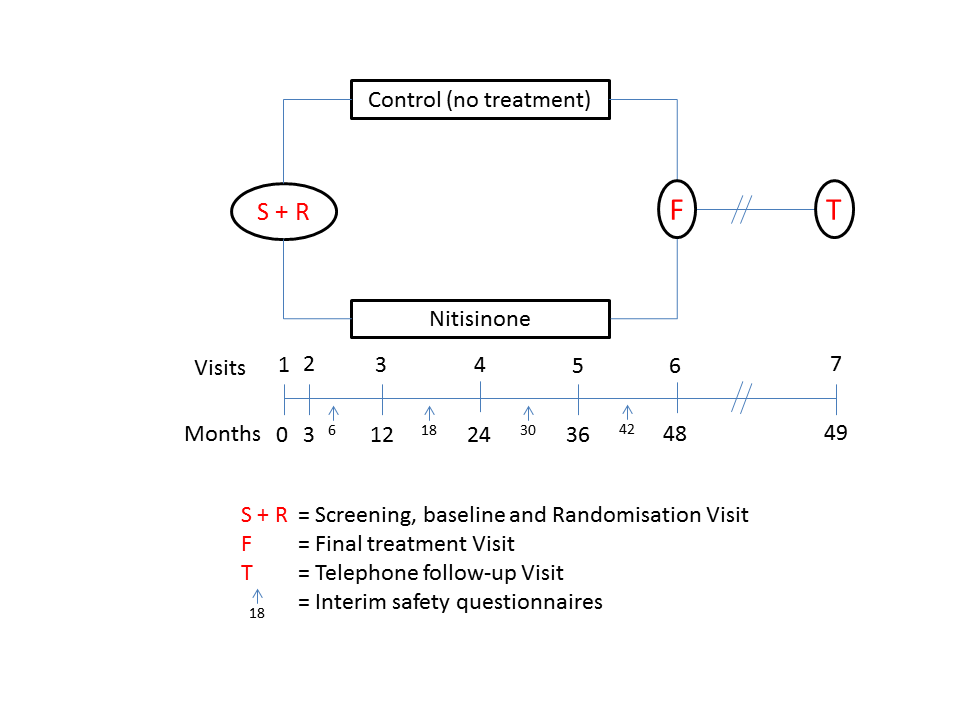
Figure S1(b).

**4.8.** Figure S2 (a-d).

a

b

c

d

**4.9.** Figure S3 (a-d).

a

b

c

d

**4.10.** Figure S4 (a-d).

a

b

c

d

**4.11.** Figure S5 (a-d).

a

b

c

d
